# Searches for scalar leptoquarks in *pp* collisions at $\varvec{\sqrt{s}}$ = 8 TeV with the ATLAS detector

**DOI:** 10.1140/epjc/s10052-015-3823-9

**Published:** 2016-01-05

**Authors:** ATLAS Collaboration, G. Aad, B. Abbott, J. Abdallah, O. Abdinov, R. Aben, M. Abolins, O. S. AbouZeid, H. Abramowicz, H. Abreu, R. Abreu, Y. Abulaiti, B. S. Acharya, L. Adamczyk, D. L. Adams, J. Adelman, S. Adomeit, T. Adye, A. A. Affolder, T. Agatonovic-Jovin, J. Agricola, J. A. Aguilar-Saavedra, S. P. Ahlen, F. Ahmadov, G. Aielli, H. Akerstedt, T. P. A. Åkesson, A. V. Akimov, G. L. Alberghi, J. Albert, S. Albrand, M. J. Alconada Verzini, M. Aleksa, I. N. Aleksandrov, C. Alexa, G. Alexander, T. Alexopoulos, M. Alhroob, G. Alimonti, L. Alio, J. Alison, S. P. Alkire, B. M. M. Allbrooke, P. P. Allport, A. Aloisio, A. Alonso, F. Alonso, C. Alpigiani, A. Altheimer, B. Alvarez Gonzalez, D. Álvarez Piqueras, M. G. Alviggi, B. T. Amadio, K. Amako, Y. Amaral Coutinho, C. Amelung, D. Amidei, S. P. Amor Dos Santos, A. Amorim, S. Amoroso, N. Amram, G. Amundsen, C. Anastopoulos, L. S. Ancu, N. Andari, T. Andeen, C. F. Anders, G. Anders, J. K. Anders, K. J. Anderson, A. Andreazza, V. Andrei, S. Angelidakis, I. Angelozzi, P. Anger, A. Angerami, F. Anghinolfi, A. V. Anisenkov, N. Anjos, A. Annovi, M. Antonelli, A. Antonov, J. Antos, F. Anulli, M. Aoki, L. Aperio Bella, G. Arabidze, Y. Arai, J. P. Araque, A. T. H. Arce, F. A. Arduh, J-F. Arguin, S. Argyropoulos, M. Arik, A. J. Armbruster, O. Arnaez, V. Arnal, H. Arnold, M. Arratia, O. Arslan, A. Artamonov, G. Artoni, S. Asai, N. Asbah, A. Ashkenazi, B. Åsman, L. Asquith, K. Assamagan, R. Astalos, M. Atkinson, N. B. Atlay, K. Augsten, M. Aurousseau, G. Avolio, B. Axen, M. K. Ayoub, G. Azuelos, M. A. Baak, A. E. Baas, M. J. Baca, C. Bacci, H. Bachacou, K. Bachas, M. Backes, M. Backhaus, P. Bagiacchi, P. Bagnaia, Y. Bai, T. Bain, J. T. Baines, O. K. Baker, E. M. Baldin, P. Balek, T. Balestri, F. Balli, E. Banas, Sw. Banerjee, A. A. E. Bannoura, H. S. Bansil, L. Barak, E. L. Barberio, D. Barberis, M. Barbero, T. Barillari, M. Barisonzi, T. Barklow, N. Barlow, S. L. Barnes, B. M. Barnett, R. M. Barnett, Z. Barnovska, A. Baroncelli, G. Barone, A. J. Barr, F. Barreiro, J. Barreiro Guimarães da Costa, R. Bartoldus, A. E. Barton, P. Bartos, A. Basalaev, A. Bassalat, A. Basye, R. L. Bates, S. J. Batista, J. R. Batley, M. Battaglia, M. Bauce, F. Bauer, H. S. Bawa, J. B. Beacham, M. D. Beattie, T. Beau, P. H. Beauchemin, R. Beccherle, P. Bechtle, H. P. Beck, K. Becker, M. Becker, M. Beckingham, C. Becot, A. J. Beddall, A. Beddall, V. A. Bednyakov, C. P. Bee, L. J. Beemster, T. A. Beermann, M. Begel, J. K. Behr, C. Belanger-Champagne, W. H. Bell, G. Bella, L. Bellagamba, A. Bellerive, M. Bellomo, K. Belotskiy, O. Beltramello, O. Benary, D. Benchekroun, M. Bender, K. Bendtz, N. Benekos, Y. Benhammou, E. Benhar Noccioli, J. A. Benitez Garcia, D. P. Benjamin, J. R. Bensinger, S. Bentvelsen, L. Beresford, M. Beretta, D. Berge, E. Bergeaas Kuutmann, N. Berger, F. Berghaus, J. Beringer, C. Bernard, N. R. Bernard, C. Bernius, F. U. Bernlochner, T. Berry, P. Berta, C. Bertella, G. Bertoli, F. Bertolucci, C. Bertsche, D. Bertsche, M. I. Besana, G. J. Besjes, O. Bessidskaia Bylund, M. Bessner, N. Besson, C. Betancourt, S. Bethke, A. J. Bevan, W. Bhimji, R. M. Bianchi, L. Bianchini, M. Bianco, O. Biebel, D. Biedermann, S. P. Bieniek, M. Biglietti, J. Bilbao De Mendizabal, H. Bilokon, M. Bindi, S. Binet, A. Bingul, C. Bini, S. Biondi, D. M. Bjergaard, C. W. Black, J. E. Black, K. M. Black, D. Blackburn, R. E. Blair, J.-B. Blanchard, J. E. Blanco, T. Blazek, I. Bloch, C. Blocker, W. Blum, U. Blumenschein, G. J. Bobbink, V. S. Bobrovnikov, S. S. Bocchetta, A. Bocci, C. Bock, M. Boehler, J. A. Bogaerts, D. Bogavac, A. G. Bogdanchikov, C. Bohm, V. Boisvert, T. Bold, V. Boldea, A. S. Boldyrev, M. Bomben, M. Bona, M. Boonekamp, A. Borisov, G. Borissov, S. Borroni, J. Bortfeldt, V. Bortolotto, K. Bos, D. Boscherini, M. Bosman, J. Boudreau, J. Bouffard, E. V. Bouhova-Thacker, D. Boumediene, C. Bourdarios, N. Bousson, S. K. Boutle, A. Boveia, J. Boyd, I. R. Boyko, I. Bozic, J. Bracinik, A. Brandt, G. Brandt, O. Brandt, U. Bratzler, B. Brau, J. E. Brau, H. M. Braun, S. F. Brazzale, W. D. Breaden Madden, K. Brendlinger, A. J. Brennan, L. Brenner, R. Brenner, S. Bressler, K. Bristow, T. M. Bristow, D. Britton, D. Britzger, F. M. Brochu, I. Brock, R. Brock, J. Bronner, G. Brooijmans, T. Brooks, W. K. Brooks, J. Brosamer, E. Brost, J. Brown, P. A. Bruckman de Renstrom, D. Bruncko, R. Bruneliere, A. Bruni, G. Bruni, M. Bruschi, N. Bruscino, L. Bryngemark, T. Buanes, Q. Buat, P. Buchholz, A. G. Buckley, S. I. Buda, I. A. Budagov, F. Buehrer, L. Bugge, M. K. Bugge, O. Bulekov, D. Bullock, H. Burckhart, S. Burdin, C. D. Burgard, B. Burghgrave, S. Burke, I. Burmeister, E. Busato, D. Büscher, V. Büscher, P. Bussey, J. M. Butler, A. I. Butt, C. M. Buttar, J. M. Butterworth, P. Butti, W. Buttinger, A. Buzatu, A. R. Buzykaev, S. CabreraUrbán, D. Caforio, V. M. Cairo, O. Cakir, N. Calace, P. Calafiura, A. Calandri, G. Calderini, P. Calfayan, L. P. Caloba, D. Calvet, S. Calvet, R. Camacho Toro, S. Camarda, P. Camarri, D. Cameron, R. Caminal Armadans, S. Campana, M. Campanelli, A. Campoverde, V. Canale, A. Canepa, M. Cano Bret, J. Cantero, R. Cantrill, T. Cao, M. D. M. Capeans Garrido, I. Caprini, M. Caprini, M. Capua, R. Caputo, R. Cardarelli, F. Cardillo, T. Carli, G. Carlino, L. Carminati, S. Caron, E. Carquin, G. D. Carrillo-Montoya, J. R. Carter, J. Carvalho, D. Casadei, M. P. Casado, M. Casolino, E. Castaneda-Miranda, A. Castelli, V. Castillo Gimenez, N. F. Castro, P. Catastini, A. Catinaccio, J. R. Catmore, A. Cattai, J. Caudron, V. Cavaliere, D. Cavalli, M. Cavalli-Sforza, V. Cavasinni, F. Ceradini, B. C. Cerio, K. Cerny, A. S. Cerqueira, A. Cerri, L. Cerrito, F. Cerutti, M. Cerv, A. Cervelli, S. A. Cetin, A. Chafaq, D. Chakraborty, I. Chalupkova, P. Chang, J. D. Chapman, D. G. Charlton, C. C. Chau, C. A. Chavez Barajas, S. Cheatham, A. Chegwidden, S. Chekanov, S. V. Chekulaev, G. A. Chelkov, M. A. Chelstowska, C. Chen, H. Chen, K. Chen, L. Chen, S. Chen, S. Chen, X. Chen, Y. Chen, H. C. Cheng, Y. Cheng, A. Cheplakov, E. Cheremushkina, R. Cherkaoui El Moursli, V. Chernyatin, E. Cheu, L. Chevalier, V. Chiarella, G. Chiarelli, G. Chiodini, A. S. Chisholm, R. T. Chislett, A. Chitan, M. V. Chizhov, K. Choi, S. Chouridou, B. K. B. Chow, V. Christodoulou, D. Chromek-Burckhart, J. Chudoba, A. J. Chuinard, J. J. Chwastowski, L. Chytka, G. Ciapetti, A. K. Ciftci, D. Cinca, V. Cindro, I. A. Cioara, A. Ciocio, F. Cirotto, Z. H. Citron, M. Ciubancan, A. Clark, B. L. Clark, P. J. Clark, R. N. Clarke, W. Cleland, C. Clement, Y. Coadou, M. Cobal, A. Coccaro, J. Cochran, L. Coffey, J. G. Cogan, L. Colasurdo, B. Cole, S. Cole, A. P. Colijn, J. Collot, T. Colombo, G. Compostella, P. Conde Muiño, E. Coniavitis, S. H. Connell, I. A. Connelly, V. Consorti, S. Constantinescu, C. Conta, G. Conti, F. Conventi, M. Cooke, B. D. Cooper, A. M. Cooper-Sarkar, T. Cornelissen, M. Corradi, F. Corriveau, A. Corso-Radu, A. Cortes-Gonzalez, G. Cortiana, G. Costa, M. J. Costa, D. Costanzo, D. Côté, G. Cottin, G. Cowan, B. E. Cox, K. Cranmer, G. Cree, S. Crépé-Renaudin, F. Crescioli, W. A. Cribbs, M. Crispin Ortuzar, M. Cristinziani, V. Croft, G. Crosetti, T. Cuhadar Donszelmann, J. Cummings, M. Curatolo, J. Cúth, C. Cuthbert, H. Czirr, P. Czodrowski, S. D’Auria, M. D’Onofrio, M. J. Da Cunha Sargedas De Sousa, C. DaVia, W. Dabrowski, A. Dafinca, T. Dai, O. Dale, F. Dallaire, C. Dallapiccola, M. Dam, J. R. Dandoy, N. P. Dang, A. C. Daniells, M. Danninger, M. Dano Hoffmann, V. Dao, G. Darbo, S. Darmora, J. Dassoulas, A. Dattagupta, W. Davey, C. David, T. Davidek, E. Davies, M. Davies, P. Davison, Y. Davygora, E. Dawe, I. Dawson, R. K. Daya-Ishmukhametova, K. De, R. de Asmundis, A. De Benedetti, S. De Castro, S. De Cecco, N. De Groot, P. de Jong, H. De la Torre, F. De Lorenzi, D. De Pedis, A. De Salvo, U. De Sanctis, A. De Santo, J. B. De Vivie De Regie, W. J. Dearnaley, R. Debbe, C. Debenedetti, D. V. Dedovich, I. Deigaard, J. Del Peso, T. Del Prete, D. Delgove, F. Deliot, C. M. Delitzsch, M. Deliyergiyev, A. Dell’Acqua, L. Dell’Asta, M. Dell’Orso, M. Della Pietra, D. della Volpe, M. Delmastro, P. A. Delsart, C. Deluca, D. A. DeMarco, S. Demers, M. Demichev, A. Demilly, S. P. Denisov, D. Derendarz, J. E. Derkaoui, F. Derue, P. Dervan, K. Desch, C. Deterre, P. O. Deviveiros, A. Dewhurst, S. Dhaliwal, A. Di Ciaccio, L. Di Ciaccio, A. Di Domenico, C. Di Donato, A. Di Girolamo, B. Di Girolamo, A. Di Mattia, B. Di Micco, R. Di Nardo, A. Di Simone, R. Di Sipio, D. Di Valentino, C. Diaconu, M. Diamond, F. A. Dias, M. A. Diaz, E. B. Diehl, J. Dietrich, S. Diglio, A. Dimitrievska, J. Dingfelder, P. Dita, S. Dita, F. Dittus, F. Djama, T. Djobava, J. I. Djuvsland, M. A. B. do Vale, D. Dobos, M. Dobre, C. Doglioni, T. Dohmae, J. Dolejsi, Z. Dolezal, B. A. Dolgoshein, M. Donadelli, S. Donati, P. Dondero, J. Donini, J. Dopke, A. Doria, M. T. Dova, A. T. Doyle, E. Drechsler, M. Dris, E. Dubreuil, E. Duchovni, G. Duckeck, O. A. Ducu, D. Duda, A. Dudarev, L. Duflot, L. Duguid, M. Dührssen, M. Dunford, H. Duran Yildiz, M. Düren, A. Durglishvili, D. Duschinger, M. Dyndal, C. Eckardt, K. M. Ecker, R. C. Edgar, W. Edson, N. C. Edwards, W. Ehrenfeld, T. Eifert, G. Eigen, K. Einsweiler, T. Ekelof, M. El Kacimi, M. Ellert, S. Elles, F. Ellinghaus, A. A. Elliot, N. Ellis, J. Elmsheuser, M. Elsing, D. Emeliyanov, Y. Enari, O. C. Endner, M. Endo, J. Erdmann, A. Ereditato, G. Ernis, J. Ernst, M. Ernst, S. Errede, E. Ertel, M. Escalier, H. Esch, C. Escobar, B. Esposito, A. I. Etienvre, E. Etzion, H. Evans, A. Ezhilov, L. Fabbri, G. Facini, R. M. Fakhrutdinov, S. Falciano, R. J. Falla, J. Faltova, Y. Fang, M. Fanti, A. Farbin, A. Farilla, T. Farooque, S. Farrell, S. M. Farrington, P. Farthouat, F. Fassi, P. Fassnacht, D. Fassouliotis, M. Faucci Giannelli, A. Favareto, L. Fayard, P. Federic, O. L. Fedin, W. Fedorko, S. Feigl, L. Feligioni, C. Feng, E. J. Feng, H. Feng, A. B. Fenyuk, L. Feremenga, P. FernandezMartinez, S. Fernandez Perez, J. Ferrando, A. Ferrari, P. Ferrari, R. Ferrari, D. E. Ferreira de Lima, A. Ferrer, D. Ferrere, C. Ferretti, A. FerrettoParodi, M. Fiascaris, F. Fiedler, A. Filipčič, M. Filipuzzi, F. Filthaut, M. Fincke-Keeler, K. D. Finelli, M. C. N. Fiolhais, L. Fiorini, A. Firan, A. Fischer, C. Fischer, J. Fischer, W. C. Fisher, E. A. Fitzgerald, N. Flaschel, I. Fleck, P. Fleischmann, S. Fleischmann, G. T. Fletcher, G. Fletcher, R. R. M. Fletcher, T. Flick, A. Floderus, L. R. Flores Castillo, M. J. Flowerdew, A. Formica, A. Forti, D. Fournier, H. Fox, S. Fracchia, P. Francavilla, M. Franchini, D. Francis, L. Franconi, M. Franklin, M. Frate, M. Fraternali, D. Freeborn, S. T. French, F. Friedrich, D. Froidevaux, J. A. Frost, C. Fukunaga, E. Fullana Torregrosa, B. G. Fulsom, T. Fusayasu, J. Fuster, C. Gabaldon, O. Gabizon, A. Gabrielli, A. Gabrielli, G. P. Gach, S. Gadatsch, S. Gadomski, G. Gagliardi, P. Gagnon, C. Galea, B. Galhardo, E. J. Gallas, B. J. Gallop, P. Gallus, G. Galster, K. K. Gan, J. Gao, Y. Gao, Y. S. Gao, F. M. GarayWalls, F. Garberson, C. García, J. E. García Navarro, M. Garcia-Sciveres, R. W. Gardner, N. Garelli, V. Garonne, C. Gatti, A. Gaudiello, G. Gaudio, B. Gaur, L. Gauthier, P. Gauzzi, I. L. Gavrilenko, C. Gay, G. Gaycken, E. N. Gazis, P. Ge, Z. Gecse, C. N. P. Gee, Ch. Geich-Gimbel, M. P. Geisler, C. Gemme, M. H. Genest, S. Gentile, M. George, S. George, D. Gerbaudo, A. Gershon, S. Ghasemi, H. Ghazlane, B. Giacobbe, S. Giagu, V. Giangiobbe, P. Giannetti, B. Gibbard, S. M. Gibson, M. Gilchriese, T. P. S. Gillam, D. Gillberg, G. Gilles, D. M. Gingrich, N. Giokaris, M. P. Giordani, F. M. Giorgi, F. M. Giorgi, P. F. Giraud, P. Giromini, D. Giugni, C. Giuliani, M. Giulini, B. K. Gjelsten, S. Gkaitatzis, I. Gkialas, E. L. Gkougkousis, L. K. Gladilin, C. Glasman, J. Glatzer, P. C. F. Glaysher, A. Glazov, M. Goblirsch-Kolb, J. R. Goddard, J. Godlewski, S. Goldfarb, T. Golling, D. Golubkov, A. Gomes, R. Gonçalo, J. Goncalves Pinto Firmino Da Costa, L. Gonella, S. González de la Hoz, G. Gonzalez Parra, S. Gonzalez-Sevilla, L. Goossens, P. A. Gorbounov, H. A. Gordon, I. Gorelov, B. Gorini, E. Gorini, A. Gorišek, E. Gornicki, A. T. Goshaw, C. Gössling, M. I. Gostkin, D. Goujdami, A. G. Goussiou, N. Govender, E. Gozani, H. M. X. Grabas, L. Graber, I. Grabowska-Bold, P. O. J. Gradin, P. Grafström, K-J. Grahn, J. Gramling, E. Gramstad, S. Grancagnolo, V. Gratchev, H. M. Gray, E. Graziani, Z. D. Greenwood, C. Grefe, K. Gregersen, I. M. Gregor, P. Grenier, J. Griffiths, A. A. Grillo, K. Grimm, S. Grinstein, Ph. Gris, J.-F. Grivaz, J. P. Grohs, A. Grohsjean, E. Gross, J. Grosse-Knetter, G. C. Grossi, Z. J. Grout, L. Guan, J. Guenther, F. Guescini, D. Guest, O. Gueta, E. Guido, T. Guillemin, S. Guindon, U. Gul, C. Gumpert, J. Guo, Y. Guo, S. Gupta, G. Gustavino, P. Gutierrez, N. G. Gutierrez Ortiz, C. Gutschow, C. Guyot, C. Gwenlan, C. B. Gwilliam, A. Haas, C. Haber, H. K. Hadavand, N. Haddad, P. Haefner, S. Hageböck, Z. Hajduk, H. Hakobyan, M. Haleem, J. Haley, D. Hall, G. Halladjian, G. D. Hallewell, K. Hamacher, P. Hamal, K. Hamano, A. Hamilton, G. N. Hamity, P. G. Hamnett, L. Han, K. Hanagaki, K. Hanawa, M. Hance, B. Haney, P. Hanke, R. Hanna, J. B. Hansen, J. D. Hansen, M. C. Hansen, P. H. Hansen, K. Hara, A. S. Hard, T. Harenberg, F. Hariri, S. Harkusha, R. D. Harrington, P. F. Harrison, F. Hartjes, M. Hasegawa, Y. Hasegawa, A. Hasib, S. Hassani, S. Haug, R. Hauser, L. Hauswald, M. Havranek, C. M. Hawkes, R. J. Hawkings, A. D. Hawkins, T. Hayashi, D. Hayden, C. P. Hays, J. M. Hays, H. S. Hayward, S. J. Haywood, S. J. Head, T. Heck, V. Hedberg, L. Heelan, S. Heim, T. Heim, B. Heinemann, L. Heinrich, J. Hejbal, L. Helary, S. Hellman, D. Hellmich, C. Helsens, J. Henderson, R. C. W. Henderson, Y. Heng, C. Hengler, S. Henkelmann, A. Henrichs, A. M. Henriques Correia, S. Henrot-Versille, G. H. Herbert, Y. Hernández Jiménez, R. Herrberg-Schubert, G. Herten, R. Hertenberger, L. Hervas, G. G. Hesketh, N. P. Hessey, J. W. Hetherly, R. Hickling, E. Higón-Rodriguez, E. Hill, J. C. Hill, K. H. Hiller, S. J. Hillier, I. Hinchliffe, E. Hines, R. R. Hinman, M. Hirose, D. Hirschbuehl, J. Hobbs, N. Hod, M. C. Hodgkinson, P. Hodgson, A. Hoecker, M. R. Hoeferkamp, F. Hoenig, M. Hohlfeld, D. Hohn, T. R. Holmes, M. Homann, T. M. Hong, L. Hooft van Huysduynen, W. H. Hopkins, Y. Horii, A. J. Horton, J-Y. Hostachy, S. Hou, A. Hoummada, J. Howard, J. Howarth, M. Hrabovsky, I. Hristova, J. Hrivnac, T. Hryn’ova, A. Hrynevich, C. Hsu, P. J. Hsu, S.-C. Hsu, D. Hu, Q. Hu, X. Hu, Y. Huang, Z. Hubacek, F. Hubaut, F. Huegging, T. B. Huffman, E. W. Hughes, G. Hughes, M. Huhtinen, T. A. Hülsing, N. Huseynov, J. Huston, J. Huth, G. Iacobucci, G. Iakovidis, I. Ibragimov, L. Iconomidou-Fayard, E. Ideal, Z. Idrissi, P. Iengo, O. Igonkina, T. Iizawa, Y. Ikegami, K. Ikematsu, M. Ikeno, Y. Ilchenko, D. Iliadis, N. Ilic, T. Ince, G. Introzzi, P. Ioannou, M. Iodice, K. Iordanidou, V. Ippolito, A. Irles Quiles, C. Isaksson, M. Ishino, M. Ishitsuka, R. Ishmukhametov, C. Issever, S. Istin, J. M. Iturbe Ponce, R. Iuppa, J. Ivarsson, W. Iwanski, H. Iwasaki, J. M. Izen, V. Izzo, S. Jabbar, B. Jackson, M. Jackson, P. Jackson, M. R. Jaekel, V. Jain, K. Jakobs, S. Jakobsen, T. Jakoubek, J. Jakubek, D. O. Jamin, D. K. Jana, E. Jansen, R. Jansky, J. Janssen, M. Janus, G. Jarlskog, N. Javadov, T. Javůrek, L. Jeanty, J. Jejelava, G.-Y. Jeng, D. Jennens, P. Jenni, J. Jentzsch, C. Jeske, S. Jézéquel, H. Ji, J. Jia, Y. Jiang, S. Jiggins, J. Jimenez Pena, S. Jin, A. Jinaru, O. Jinnouchi, M. D. Joergensen, P. Johansson, K. A. Johns, K. Jon-And, G. Jones, R. W. L. Jones, T. J. Jones, J. Jongmanns, P. M. Jorge, K. D. Joshi, J. Jovicevic, X. Ju, C. A. Jung, P. Jussel, A. Juste Rozas, M. Kaci, A. Kaczmarska, M. Kado, H. Kagan, M. Kagan, S. J. Kahn, E. Kajomovitz, C. W. Kalderon, S. Kama, A. Kamenshchikov, N. Kanaya, S. Kaneti, V. A. Kantserov, J. Kanzaki, B. Kaplan, L. S. Kaplan, A. Kapliy, D. Kar, K. Karakostas, A. Karamaoun, N. Karastathis, M. J. Kareem, E. Karentzos, M. Karnevskiy, S. N. Karpov, Z. M. Karpova, K. Karthik, V. Kartvelishvili, A. N. Karyukhin, K. Kasahara, L. Kashif, R. D. Kass, A. Kastanas, Y. Kataoka, C. Kato, A. Katre, J. Katzy, K. Kawagoe, T. Kawamoto, G. Kawamura, S. Kazama, V. F. Kazanin, R. Keeler, R. Kehoe, J. S. Keller, J. J. Kempster, H. Keoshkerian, O. Kepka, B. P. Kerševan, S. Kersten, R. A. Keyes, F. Khalil-zada, H. Khandanyan, A. Khanov, A. G. Kharlamov, T. J. Khoo, V. Khovanskiy, E. Khramov, J. Khubua, S. Kido, H. Y. Kim, S. H. Kim, Y. K. Kim, N. Kimura, O. M. Kind, B. T. King, M. King, S. B. King, J. Kirk, A. E. Kiryunin, T. Kishimoto, D. Kisielewska, F. Kiss, K. Kiuchi, O. Kivernyk, E. Kladiva, M. H. Klein, M. Klein, U. Klein, K. Kleinknecht, P. Klimek, A. Klimentov, R. Klingenberg, J. A. Klinger, T. Klioutchnikova, E.-E. Kluge, P. Kluit, S. Kluth, J. Knapik, E. Kneringer, E. B. F. G. Knoops, A. Knue, A. Kobayashi, D. Kobayashi, T. Kobayashi, M. Kobel, M. Kocian, P. Kodys, T. Koffas, E. Koffeman, L. A. Kogan, S. Kohlmann, Z. Kohout, T. Kohriki, T. Koi, H. Kolanoski, I. Koletsou, A. A. Komar, Y. Komori, T. Kondo, N. Kondrashova, K. Köneke, A. C. König, T. Kono, R. Konoplich, N. Konstantinidis, R. Kopeliansky, S. Koperny, L. Köpke, A. K. Kopp, K. Korcyl, K. Kordas, A. Korn, A. A. Korol, I. Korolkov, E. V. Korolkova, O. Kortner, S. Kortner, T. Kosek, V. V. Kostyukhin, V. M. Kotov, A. Kotwal, A. Kourkoumeli-Charalampidi, C. Kourkoumelis, V. Kouskoura, A. Koutsman, R. Kowalewski, T. Z. Kowalski, W. Kozanecki, A. S. Kozhin, V. A. Kramarenko, G. Kramberger, D. Krasnopevtsev, M. W. Krasny, A. Krasznahorkay, J. K. Kraus, A. Kravchenko, S. Kreiss, M. Kretz, J. Kretzschmar, K. Kreutzfeldt, P. Krieger, K. Krizka, K. Kroeninger, H. Kroha, J. Kroll, J. Kroseberg, J. Krstic, U. Kruchonak, H. Krüger, N. Krumnack, A. Kruse, M. C. Kruse, M. Kruskal, T. Kubota, H. Kucuk, S. Kuday, S. Kuehn, A. Kugel, F. Kuger, A. Kuhl, T. Kuhl, V. Kukhtin, R. Kukla, Y. Kulchitsky, S. Kuleshov, M. Kuna, T. Kunigo, A. Kupco, H. Kurashige, Y. A. Kurochkin, V. Kus, E. S. Kuwertz, M. Kuze, J. Kvita, T. Kwan, D. Kyriazopoulos, A. LaRosa, J. L. La RosaNavarro, L. La Rotonda, C. Lacasta, F. Lacava, J. Lacey, H. Lacker, D. Lacour, V. R. Lacuesta, E. Ladygin, R. Lafaye, B. Laforge, T. Lagouri, S. Lai, L. Lambourne, S. Lammers, C. L. Lampen, W. Lampl, E. Lançon, U. Landgraf, M. P. J. Landon, V. S. Lang, J. C. Lange, A. J. Lankford, F. Lanni, K. Lantzsch, A. Lanza, S. Laplace, C. Lapoire, J. F. Laporte, T. Lari, F. Lasagni Manghi, M. Lassnig, P. Laurelli, W. Lavrijsen, A. T. Law, P. Laycock, T. Lazovich, O. Le Dortz, E. Le Guirriec, E. Le Menedeu, M. LeBlanc, T. Le Compte, F. Ledroit-Guillon, C. A. Lee, S. C. Lee, L. Lee, G. Lefebvre, M. Lefebvre, F. Legger, C. Leggett, A. Lehan, G. Lehmann Miotto, X. Lei, W. A. Leight, A. Leisos, A. G. Leister, M. A. L. Leite, R. Leitner, D. Lellouch, B. Lemmer, K. J. C. Leney, T. Lenz, B. Lenzi, R. Leone, S. Leone, C. Leonidopoulos, S. Leontsinis, C. Leroy, C. G. Lester, M. Levchenko, J. Levêque, D. Levin, L. J. Levinson, M. Levy, A. Lewis, A. M. Leyko, M. Leyton, B. Li, H. Li, H. L. Li, L. Li, L. Li, S. Li, X. Li, Y. Li, Z. Liang, H. Liao, B. Liberti, A. Liblong, P. Lichard, K. Lie, J. Liebal, W. Liebig, C. Limbach, A. Limosani, S. C. Lin, T. H. Lin, F. Linde, B. E. Lindquist, J. T. Linnemann, E. Lipeles, A. Lipniacka, M. Lisovyi, T. M. Liss, D. Lissauer, A. Lister, A. M. Litke, B. Liu, D. Liu, H. Liu, J. Liu, J. B. Liu, K. Liu, L. Liu, M. Liu, M. Liu, Y. Liu, M. Livan, A. Lleres, J. Llorente Merino, S. L. Lloyd, F. Lo Sterzo, E. Lobodzinska, P. Loch, W. S. Lockman, F. K. Loebinger, A. E. Loevschall-Jensen, K. M. Loew, A. Loginov, T. Lohse, K. Lohwasser, M. Lokajicek, B. A. Long, J. D. Long, R. E. Long, K. A. Looper, L. Lopes, D. Lopez Mateos, B. Lopez Paredes, I. Lopez Paz, J. Lorenz, N. Lorenzo Martinez, M. Losada, P. J. Lösel, X. Lou, A. Lounis, J. Love, P. A. Love, N. Lu, H. J. Lubatti, C. Luci, A. Lucotte, F. Luehring, W. Lukas, L. Luminari, O. Lundberg, B. Lund-Jensen, D. Lynn, R. Lysak, E. Lytken, H. Ma, L. L. Ma, G. Maccarrone, A. Macchiolo, C. M. Macdonald, B. Maček, J. Machado Miguens, D. Macina, D. Madaffari, R. Madar, H. J. Maddocks, W. F. Mader, A. Madsen, J. Maeda, S. Maeland, T. Maeno, A. Maevskiy, E. Magradze, K. Mahboubi, J. Mahlstedt, C. Maiani, C. Maidantchik, A. A. Maier, T. Maier, A. Maio, S. Majewski, Y. Makida, N. Makovec, B. Malaescu, Pa. Malecki, V. P. Maleev, F. Malek, U. Mallik, D. Malon, C. Malone, S. Maltezos, V. M. Malyshev, S. Malyukov, J. Mamuzic, G. Mancini, B. Mandelli, L. Mandelli, I. Mandić, R. Mandrysch, J. Maneira, A. Manfredini, L. Manhaes de AndradeFilho, J. Manjarres Ramos, A. Mann, A. Manousakis-Katsikakis, B. Mansoulie, R. Mantifel, M. Mantoani, L. Mapelli, L. March, G. Marchiori, M. Marcisovsky, C. P. Marino, M. Marjanovic, D. E. Marley, F. Marroquim, S. P. Marsden, Z. Marshall, L. F. Marti, S. Marti-Garcia, B. Martin, T. A. Martin, V. J. Martin, B. Martin dit Latour, M. Martinez, S. Martin-Haugh, V. S. Martoiu, A. C. Martyniuk, M. Marx, F. Marzano, A. Marzin, L. Masetti, T. Mashimo, R. Mashinistov, J. Masik, A. L. Maslennikov, I. Massa, L. Massa, P. Mastrandrea, A. Mastroberardino, T. Masubuchi, P. Mättig, J. Mattmann, J. Maurer, S. J. Maxfield, D. A. Maximov, R. Mazini, S. M. Mazza, L. Mazzaferro, G. McGoldrick, S. P. Mc Kee, A. McCarn, R. L. McCarthy, T. G. McCarthy, N. A. McCubbin, K. W. McFarlane, J. A. Mcfayden, G. Mchedlidze, S. J. McMahon, R. A. McPherson, M. Medinnis, S. Meehan, S. Mehlhase, A. Mehta, K. Meier, C. Meineck, B. Meirose, B. R. Mellado Garcia, F. Meloni, A. Mengarelli, S. Menke, E. Meoni, K. M. Mercurio, S. Mergelmeyer, P. Mermod, L. Merola, C. Meroni, F. S. Merritt, A. Messina, J. Metcalfe, A. S. Mete, C. Meyer, C. Meyer, J-P. Meyer, J. Meyer, H. Meyer Zu Theenhausen, R. P. Middleton, S. Miglioranzi, L. Mijović, G. Mikenberg, M. Mikestikova, M. Mikuž, M. Milesi, A. Milic, D. W. Miller, C. Mills, A. Milov, D. A. Milstead, A. A. Minaenko, Y. Minami, I. A. Minashvili, A. I. Mincer, B. Mindur, M. Mineev, Y. Ming, L. M. Mir, K. P. Mistry, T. Mitani, J. Mitrevski, V. A. Mitsou, A. Miucci, P. S. Miyagawa, J. U. Mjörnmark, T. Moa, K. Mochizuki, S. Mohapatra, W. Mohr, S. Molander, R. Moles-Valls, R. Monden, K. Mönig, C. Monini, J. Monk, E. Monnier, J. MontejoBerlingen, F. Monticelli, S. Monzani, R. W. Moore, N. Morange, D. Moreno, M. MorenoLlácer, P. Morettini, D. Mori, T. Mori, M. Morii, M. Morinaga, V. Morisbak, S. Moritz, A. K. Morley, G. Mornacchi, J. D. Morris, S. S. Mortensen, A. Morton, L. Morvaj, M. Mosidze, J. Moss, K. Motohashi, R. Mount, E. Mountricha, S. V. Mouraviev, E. J. W. Moyse, S. Muanza, R. D. Mudd, F. Mueller, J. Mueller, R. S. P. Mueller, T. Mueller, D. Muenstermann, P. Mullen, G. A. Mullier, J. A. Murillo Quijada, W. J. Murray, H. Musheghyan, E. Musto, A. G. Myagkov, M. Myska, B. P. Nachman, O. Nackenhorst, J. Nadal, K. Nagai, R. Nagai, Y. Nagai, K. Nagano, A. Nagarkar, Y. Nagasaka, K. Nagata, M. Nagel, E. Nagy, A. M. Nairz, Y. Nakahama, K. Nakamura, T. Nakamura, I. Nakano, H. Namasivayam, R. F. Naranjo Garcia, R. Narayan, D. I. Narrias Villar, T. Naumann, G. Navarro, R. Nayyar, H. A. Neal, P. Yu. Nechaeva, T. J. Neep, P. D. Nef, A. Negri, M. Negrini, S. Nektarijevic, C. Nellist, A. Nelson, S. Nemecek, P. Nemethy, A. A. Nepomuceno, M. Nessi, M. S. Neubauer, M. Neumann, R. M. Neves, P. Nevski, P. R. Newman, D. H. Nguyen, R. B. Nickerson, R. Nicolaidou, B. Nicquevert, J. Nielsen, N. Nikiforou, A. Nikiforov, V. Nikolaenko, I. Nikolic-Audit, K. Nikolopoulos, J. K. Nilsen, P. Nilsson, Y. Ninomiya, A. Nisati, R. Nisius, T. Nobe, M. Nomachi, I. Nomidis, T. Nooney, S. Norberg, M. Nordberg, O. Novgorodova, S. Nowak, M. Nozaki, L. Nozka, K. Ntekas, G. Nunes Hanninger, T. Nunnemann, E. Nurse, F. Nuti, B. J. O’Brien, F. O’grady, D. C. O’Neil, V. O’Shea, F. G. Oakham, H. Oberlack, T. Obermann, J. Ocariz, A. Ochi, I. Ochoa, J. P. Ochoa-Ricoux, S. Oda, S. Odaka, H. Ogren, A. Oh, S. H. Oh, C. C. Ohm, H. Ohman, H. Oide, W. Okamura, H. Okawa, Y. Okumura, T. Okuyama, A. Olariu, S. A. Olivares Pino, D. Oliveira Damazio, E. Oliver Garcia, A. Olszewski, J. Olszowska, A. Onofre, K. Onogi, P. U. E. Onyisi, C. J. Oram, M. J. Oreglia, Y. Oren, D. Orestano, N. Orlando, C. Oropeza Barrera, R. S. Orr, B. Osculati, R. Ospanov, G. Otero y Garzon, H. Otono, M. Ouchrif, F. Ould-Saada, A. Ouraou, K. P. Oussoren, Q. Ouyang, A. Ovcharova, M. Owen, R. E. Owen, V. E. Ozcan, N. Ozturk, K. Pachal, A. PachecoPages, C. Padilla Aranda, M. Pagáčová, S. Pagan Griso, E. Paganis, F. Paige, P. Pais, K. Pajchel, G. Palacino, S. Palestini, M. Palka, D. Pallin, A. Palma, Y. B. Pan, E. Panagiotopoulou, C. E. Pandini, J. G. Panduro Vazquez, P. Pani, S. Panitkin, D. Pantea, L. Paolozzi, Th. D. Papadopoulou, K. Papageorgiou, A. Paramonov, D. Paredes Hernandez, M. A. Parker, K. A. Parker, F. Parodi, J. A. Parsons, U. Parzefall, E. Pasqualucci, S. Passaggio, F. Pastore, Fr. Pastore, G. Pásztor, S. Pataraia, N. D. Patel, J. R. Pater, T. Pauly, J. Pearce, B. Pearson, L. E. Pedersen, M. Pedersen, S. Pedraza Lopez, R. Pedro, S. V. Peleganchuk, D. Pelikan, O. Penc, C. Peng, H. Peng, B. Penning, J. Penwell, D. V. Perepelitsa, E. Perez Codina, M. T. PérezGarcía-Estañ, L. Perini, H. Pernegger, S. Perrella, R. Peschke, V. D. Peshekhonov, K. Peters, R. F. Y. Peters, B. A. Petersen, T. C. Petersen, E. Petit, A. Petridis, C. Petridou, P. Petroff, E. Petrolo, F. Petrucci, N. E. Pettersson, R. Pezoa, P. W. Phillips, G. Piacquadio, E. Pianori, A. Picazio, E. Piccaro, M. Piccinini, M. A. Pickering, R. Piegaia, D. T. Pignotti, J. E. Pilcher, A. D. Pilkington, J. Pina, M. Pinamonti, J. L. Pinfold, A. Pingel, S. Pires, H. Pirumov, M. Pitt, C. Pizio, L. Plazak, M.-A. Pleier, V. Pleskot, E. Plotnikova, P. Plucinski, D. Pluth, R. Poettgen, L. Poggioli, D. Pohl, G. Polesello, A. Poley, A. Policicchio, R. Polifka, A. Polini, C. S. Pollard, V. Polychronakos, K. Pommès, L. Pontecorvo, B. G. Pope, G. A. Popeneciu, D. S. Popovic, A. Poppleton, S. Pospisil, K. Potamianos, I. N. Potrap, C. J. Potter, C. T. Potter, G. Poulard, J. Poveda, V. Pozdnyakov, P. Pralavorio, A. Pranko, S. Prasad, S. Prell, D. Price, L. E. Price, M. Primavera, S. Prince, M. Proissl, K. Prokofiev, F. Prokoshin, E. Protopapadaki, S. Protopopescu, J. Proudfoot, M. Przybycien, E. Ptacek, D. Puddu, E. Pueschel, D. Puldon, M. Purohit, P. Puzo, J. Qian, G. Qin, Y. Qin, A. Quadt, D. R. Quarrie, W. B. Quayle, M. Queitsch-Maitland, D. Quilty, S. Raddum, V. Radeka, V. Radescu, S. K. Radhakrishnan, P. Radloff, P. Rados, F. Ragusa, G. Rahal, S. Rajagopalan, M. Rammensee, C. Rangel-Smith, F. Rauscher, S. Rave, T. Ravenscroft, M. Raymond, A. L. Read, N. P. Readioff, D. M. Rebuzzi, A. Redelbach, G. Redlinger, R. Reece, K. Reeves, L. Rehnisch, J. Reichert, H. Reisin, M. Relich, C. Rembser, H. Ren, A. Renaud, M. Rescigno, S. Resconi, O. L. Rezanova, P. Reznicek, R. Rezvani, R. Richter, S. Richter, E. Richter-Was, O. Ricken, M. Ridel, P. Rieck, C. J. Riegel, J. Rieger, O. Rifki, M. Rijssenbeek, A. Rimoldi, L. Rinaldi, B. Ristić, E. Ritsch, I. Riu, F. Rizatdinova, E. Rizvi, S. H. Robertson, A. Robichaud-Veronneau, D. Robinson, J. E. M. Robinson, A. Robson, C. Roda, S. Roe, O. Røhne, S. Rolli, A. Romaniouk, M. Romano, S. M. Romano Saez, E. Romero Adam, N. Rompotis, M. Ronzani, L. Roos, E. Ros, S. Rosati, K. Rosbach, P. Rose, P. L. Rosendahl, O. Rosenthal, V. Rossetti, E. Rossi, L. P. Rossi, J. H. N. Rosten, R. Rosten, M. Rotaru, I. Roth, J. Rothberg, D. Rousseau, C. R. Royon, A. Rozanov, Y. Rozen, X. Ruan, F. Rubbo, I. Rubinskiy, V. I. Rud, C. Rudolph, M. S. Rudolph, F. Rühr, A. Ruiz-Martinez, Z. Rurikova, N. A. Rusakovich, A. Ruschke, H. L. Russell, J. P. Rutherfoord, N. Ruthmann, Y. F. Ryabov, M. Rybar, G. Rybkin, N. C. Ryder, A. F. Saavedra, G. Sabato, S. Sacerdoti, A. Saddique, H. F-W. Sadrozinski, R. Sadykov, F. Safai Tehrani, M. Sahinsoy, M. Saimpert, T. Saito, H. Sakamoto, Y. Sakurai, G. Salamanna, A. Salamon, J. E. SalazarLoyola, M. Saleem, D. Salek, P. H. Sales DeBruin, D. Salihagic, A. Salnikov, J. Salt, D. Salvatore, F. Salvatore, A. Salvucci, A. Salzburger, D. Sammel, D. Sampsonidis, A. Sanchez, J. Sánchez, V. SanchezMartinez, H. Sandaker, R. L. Sandbach, H. G. Sander, M. P. Sanders, M. Sandhoff, C. Sandoval, R. Sandstroem, D. P. C. Sankey, M. Sannino, A. Sansoni, C. Santoni, R. Santonico, H. Santos, I. SantoyoCastillo, K. Sapp, A. Sapronov, J. G. Saraiva, B. Sarrazin, O. Sasaki, Y. Sasaki, K. Sato, G. Sauvage, E. Sauvan, G. Savage, P. Savard, C. Sawyer, L. Sawyer, J. Saxon, C. Sbarra, A. Sbrizzi, T. Scanlon, D. A. Scannicchio, M. Scarcella, V. Scarfone, J. Schaarschmidt, P. Schacht, D. Schaefer, R. Schaefer, J. Schaeffer, S. Schaepe, S. Schaetzel, U. Schäfer, A. C. Schaffer, D. Schaile, R. D. Schamberger, V. Scharf, V. A. Schegelsky, D. Scheirich, M. Schernau, C. Schiavi, C. Schillo, M. Schioppa, S. Schlenker, K. Schmieden, C. Schmitt, S. Schmitt, S. Schmitt, B. Schneider, Y. J. Schnellbach, U. Schnoor, L. Schoeffel, A. Schoening, B. D. Schoenrock, E. Schopf, A. L. S. Schorlemmer, M. Schott, D. Schouten, J. Schovancova, S. Schramm, M. Schreyer, C. Schroeder, N. Schuh, M. J. Schultens, H.-C. Schultz-Coulon, H. Schulz, M. Schumacher, B. A. Schumm, Ph. Schune, C. Schwanenberger, A. Schwartzman, T. A. Schwarz, Ph. Schwegler, H. Schweiger, Ph. Schwemling, R. Schwienhorst, J. Schwindling, T. Schwindt, F. G. Sciacca, E. Scifo, G. Sciolla, F. Scuri, F. Scutti, J. Searcy, G. Sedov, E. Sedykh, P. Seema, S. C. Seidel, A. Seiden, F. Seifert, J. M. Seixas, G. Sekhniaidze, K. Sekhon, S. J. Sekula, D. M. Seliverstov, N. Semprini-Cesari, C. Serfon, L. Serin, L. Serkin, T. Serre, M. Sessa, R. Seuster, H. Severini, T. Sfiligoj, F. Sforza, A. Sfyrla, E. Shabalina, M. Shamim, L. Y. Shan, R. Shang, J. T. Shank, M. Shapiro, P. B. Shatalov, K. Shaw, S. M. Shaw, A. Shcherbakova, C. Y. Shehu, P. Sherwood, L. Shi, S. Shimizu, C. O. Shimmin, M. Shimojima, M. Shiyakova, A. Shmeleva, D. Shoaleh Saadi, M. J. Shochet, S. Shojaii, S. Shrestha, E. Shulga, M. A. Shupe, S. Shushkevich, P. Sicho, P. E. Sidebo, O. Sidiropoulou, D. Sidorov, A. Sidoti, F. Siegert, Dj. Sijacki, J. Silva, Y. Silver, S. B. Silverstein, V. Simak, O. Simard, Lj. Simic, S. Simion, E. Simioni, B. Simmons, D. Simon, P. Sinervo, N. B. Sinev, M. Sioli, G. Siragusa, A. N. Sisakyan, S. Yu. Sivoklokov, J. Sjölin, T. B. Sjursen, M. B. Skinner, H. P. Skottowe, P. Skubic, M. Slater, T. Slavicek, M. Slawinska, K. Sliwa, V. Smakhtin, B. H. Smart, L. Smestad, S. Yu. Smirnov, Y. Smirnov, L. N. Smirnova, O. Smirnova, M. N. K. Smith, R. W. Smith, M. Smizanska, K. Smolek, A. A. Snesarev, G. Snidero, S. Snyder, R. Sobie, F. Socher, A. Soffer, D. A. Soh, G. Sokhrannyi, C. A. Solans, M. Solar, J. Solc, E. Yu. Soldatov, U. Soldevila, A. A. Solodkov, A. Soloshenko, O. V. Solovyanov, V. Solovyev, P. Sommer, H. Y. Song, N. Soni, A. Sood, A. Sopczak, B. Sopko, V. Sopko, V. Sorin, D. Sosa, M. Sosebee, C. L. Sotiropoulou, R. Soualah, A. M. Soukharev, D. South, B. C. Sowden, S. Spagnolo, M. Spalla, M. Spangenberg, F. Spanò, W. R. Spearman, D. Sperlich, F. Spettel, R. Spighi, G. Spigo, L. A. Spiller, M. Spousta, T. Spreitzer, R. D. St. Denis, A. Stabile, S. Staerz, J. Stahlman, R. Stamen, S. Stamm, E. Stanecka, C. Stanescu, M. Stanescu-Bellu, M. M. Stanitzki, S. Stapnes, E. A. Starchenko, J. Stark, P. Staroba, P. Starovoitov, R. Staszewski, P. Steinberg, B. Stelzer, H. J. Stelzer, O. Stelzer-Chilton, H. Stenzel, G. A. Stewart, J. A. Stillings, M. C. Stockton, M. Stoebe, G. Stoicea, P. Stolte, S. Stonjek, A. R. Stradling, A. Straessner, M. E. Stramaglia, J. Strandberg, S. Strandberg, A. Strandlie, E. Strauss, M. Strauss, P. Strizenec, R. Ströhmer, D. M. Strom, R. Stroynowski, A. Strubig, S. A. Stucci, B. Stugu, N. A. Styles, D. Su, J. Su, R. Subramaniam, A. Succurro, Y. Sugaya, M. Suk, V. V. Sulin, S. Sultansoy, T. Sumida, S. Sun, X. Sun, J. E. Sundermann, K. Suruliz, G. Susinno, M. R. Sutton, S. Suzuki, M. Svatos, M. Swiatlowski, I. Sykora, T. Sykora, D. Ta, C. Taccini, K. Tackmann, J. Taenzer, A. Taffard, R. Tafirout, N. Taiblum, H. Takai, R. Takashima, H. Takeda, T. Takeshita, Y. Takubo, M. Talby, A. A. Talyshev, J. Y. C. Tam, K. G. Tan, J. Tanaka, R. Tanaka, S. Tanaka, B. B. Tannenwald, N. Tannoury, S. Tapprogge, S. Tarem, F. Tarrade, G. F. Tartarelli, P. Tas, M. Tasevsky, T. Tashiro, E. Tassi, A. Tavares Delgado, Y. Tayalati, F. E. Taylor, G. N. Taylor, P. T. E. Taylor, W. Taylor, F. A. Teischinger, M. Teixeira Dias Castanheira, P. Teixeira-Dias, K. K. Temming, D. Temple, H. Ten Kate, P. K. Teng, J. J. Teoh, F. Tepel, S. Terada, K. Terashi, J. Terron, S. Terzo, M. Testa, R. J. Teuscher, T. Theveneaux-Pelzer, J. P. Thomas, J. Thomas-Wilsker, E. N. Thompson, P. D. Thompson, R. J. Thompson, A. S. Thompson, L. A. Thomsen, E. Thomson, M. Thomson, R. P. Thun, M. J. Tibbetts, R. E. TicseTorres, V. O. Tikhomirov, Yu. A. Tikhonov, S. Timoshenko, E. Tiouchichine, P. Tipton, S. Tisserant, K. Todome, T. Todorov, S. Todorova-Nova, J. Tojo, S. Tokár, K. Tokushuku, K. Tollefson, E. Tolley, L. Tomlinson, M. Tomoto, L. Tompkins, K. Toms, E. Torrence, H. Torres, E. Torró Pastor, J. Toth, F. Touchard, D. R. Tovey, T. Trefzger, L. Tremblet, A. Tricoli, I. M. Trigger, S. Trincaz-Duvoid, M. F. Tripiana, W. Trischuk, B. Trocmé, C. Troncon, M. Trottier-McDonald, M. Trovatelli, L. Truong, M. Trzebinski, A. Trzupek, C. Tsarouchas, J. C-L. Tseng, P. V. Tsiareshka, D. Tsionou, G. Tsipolitis, N. Tsirintanis, S. Tsiskaridze, V. Tsiskaridze, E. G. Tskhadadze, I. I. Tsukerman, V. Tsulaia, S. Tsuno, D. Tsybychev, A. Tudorache, V. Tudorache, A. N. Tuna, S. A. Tupputi, S. Turchikhin, D. Turecek, R. Turra, A. J. Turvey, P. M. Tuts, A. Tykhonov, M. Tylmad, M. Tyndel, I. Ueda, R. Ueno, M. Ughetto, M. Ugland, F. Ukegawa, G. Unal, A. Undrus, G. Unel, F. C. Ungaro, Y. Unno, C. Unverdorben, J. Urban, P. Urquijo, P. Urrejola, G. Usai, A. Usanova, L. Vacavant, V. Vacek, B. Vachon, C. Valderanis, N. Valencic, S. Valentinetti, A. Valero, L. Valery, S. Valkar, E. Valladolid Gallego, S. Vallecorsa, J. A. Valls Ferrer, W. Van Den Wollenberg, P. C. Van Der Deijl, R. van der Geer, H. van der Graaf, N. van Eldik, P. van Gemmeren, J. Van Nieuwkoop, I. van Vulpen, M. C. van Woerden, M. Vanadia, W. Vandelli, R. Vanguri, A. Vaniachine, F. Vannucci, G. Vardanyan, R. Vari, E. W. Varnes, T. Varol, D. Varouchas, A. Vartapetian, K. E. Varvell, F. Vazeille, T. Vazquez Schroeder, J. Veatch, L. M. Veloce, F. Veloso, T. Velz, S. Veneziano, A. Ventura, D. Ventura, M. Venturi, N. Venturi, A. Venturini, V. Vercesi, M. Verducci, W. Verkerke, J. C. Vermeulen, A. Vest, M. C. Vetterli, O. Viazlo, I. Vichou, T. Vickey, O. E. VickeyBoeriu, G. H. A. Viehhauser, S. Viel, R. Vigne, M. Villa, M. Villaplana Perez, E. Vilucchi, M. G. Vincter, V. B. Vinogradov, I. Vivarelli, F. Vives Vaque, S. Vlachos, D. Vladoiu, M. Vlasak, M. Vogel, P. Vokac, G. Volpi, M. Volpi, H. von der Schmitt, H. von Radziewski, E. von Toerne, V. Vorobel, K. Vorobev, M. Vos, R. Voss, J. H. Vossebeld, N. Vranjes, M. Vranjes Milosavljevic, V. Vrba, M. Vreeswijk, R. Vuillermet, I. Vukotic, Z. Vykydal, P. Wagner, W. Wagner, H. Wahlberg, S. Wahrmund, J. Wakabayashi, J. Walder, R. Walker, W. Walkowiak, C. Wang, F. Wang, H. Wang, H. Wang, J. Wang, J. Wang, K. Wang, R. Wang, S. M. Wang, T. Wang, T. Wang, X. Wang, C. Wanotayaroj, A. Warburton, C. P. Ward, D. R. Wardrope, A. Washbrook, C. Wasicki, P. M. Watkins, A. T. Watson, I. J. Watson, M. F. Watson, G. Watts, S. Watts, B. M. Waugh, S. Webb, M. S. Weber, S. W. Weber, J. S. Webster, A. R. Weidberg, B. Weinert, J. Weingarten, C. Weiser, H. Weits, P. S. Wells, T. Wenaus, T. Wengler, S. Wenig, N. Wermes, M. Werner, P. Werner, M. Wessels, J. Wetter, K. Whalen, A. M. Wharton, A. White, M. J. White, R. White, S. White, D. Whiteson, F. J. Wickens, W. Wiedenmann, M. Wielers, P. Wienemann, C. Wiglesworth, L. A. M. Wiik-Fuchs, A. Wildauer, H. G. Wilkens, H. H. Williams, S. Williams, C. Willis, S. Willocq, A. Wilson, J. A. Wilson, I. Wingerter-Seez, F. Winklmeier, B. T. Winter, M. Wittgen, J. Wittkowski, S. J. Wollstadt, M. W. Wolter, H. Wolters, B. K. Wosiek, J. Wotschack, M. J. Woudstra, K. W. Wozniak, M. Wu, M. Wu, S. L. Wu, X. Wu, Y. Wu, T. R. Wyatt, B. M. Wynne, S. Xella, D. Xu, L. Xu, B. Yabsley, S. Yacoob, R. Yakabe, M. Yamada, D. Yamaguchi, Y. Yamaguchi, A. Yamamoto, S. Yamamoto, T. Yamanaka, K. Yamauchi, Y. Yamazaki, Z. Yan, H. Yang, H. Yang, Y. Yang, W-M. Yao, Y. Yasu, E. Yatsenko, K. H. Yau Wong, J. Ye, S. Ye, I. Yeletskikh, A. L. Yen, E. Yildirim, K. Yorita, R. Yoshida, K. Yoshihara, C. Young, C. J. S. Young, S. Youssef, D. R. Yu, J. Yu, J. M. Yu, J. Yu, L. Yuan, S. P. Y. Yuen, A. Yurkewicz, I. Yusuff, B. Zabinski, R. Zaidan, A. M. Zaitsev, J. Zalieckas, A. Zaman, S. Zambito, L. Zanello, D. Zanzi, C. Zeitnitz, M. Zeman, A. Zemla, Q. Zeng, K. Zengel, O. Zenin, T. Ženiš, D. Zerwas, D. Zhang, F. Zhang, H. Zhang, J. Zhang, L. Zhang, R. Zhang, X. Zhang, Z. Zhang, X. Zhao, Y. Zhao, Z. Zhao, A. Zhemchugov, J. Zhong, B. Zhou, C. Zhou, L. Zhou, L. Zhou, M. Zhou, N. Zhou, C. G. Zhu, H. Zhu, J. Zhu, Y. Zhu, X. Zhuang, K. Zhukov, A. Zibell, D. Zieminska, N. I. Zimine, C. Zimmermann, S. Zimmermann, Z. Zinonos, M. Zinser, M. Ziolkowski, L. Živković, G. Zobernig, A. Zoccoli, M. zur Nedden, G. Zurzolo, L. Zwalinski

**Affiliations:** Department of Physics, University of Adelaide, Adelaide, Australia; Physics Department, SUNY Albany, Albany, NY USA; Department of Physics, University of Alberta, Edmonton, AB Canada; Department of Physics, Ankara University, Ankara, Turkey; Istanbul Aydin University, Istanbul, Turkey; Division of Physics, TOBB University of Economics and Technology, Ankara, Turkey; LAPP, CNRS/IN2P3 and Université Savoie Mont Blanc, Annecy-le-Vieux, France; High Energy Physics Division, Argonne National Laboratory, Argonne, IL USA; Department of Physics, University of Arizona, Tucson, AZ USA; Department of Physics, The University of Texas at Arlington, Arlington, TX USA; Physics Department, University of Athens, Athens, Greece; Physics Department, National Technical University of Athens, Zografou, Greece; Institute of Physics, Azerbaijan Academy of Sciences, Baku, Azerbaijan; Institut de Física d’Altes Energies and Departament de Física de la Universitat Autònoma de Barcelona, Barcelona, Spain; Institute of Physics, University of Belgrade, Belgrade, Serbia; Department for Physics and Technology, University of Bergen, Bergen, Norway; Physics Division, Lawrence Berkeley National Laboratory and University of California, Berkeley, CA USA; Department of Physics, Humboldt University, Berlin, Germany; Albert Einstein Center for Fundamental Physics and Laboratory for High Energy Physics, University of Bern, Bern, Switzerland; School of Physics and Astronomy, University of Birmingham, Birmingham, UK; Department of Physics, Bogazici University, Istanbul, Turkey; Department of Physics Engineering, Gaziantep University, Gaziantep, Turkey; Department of Physics, Dogus University, Istanbul, Turkey; INFN Sezione di Bologna, Bologna, Italy; Dipartimento di Fisica e Astronomia, Università di Bologna, Bologna, Italy; Physikalisches Institut, University of Bonn, Bonn, Germany; Department of Physics, Boston University, Boston, MA USA; Department of Physics, Brandeis University, Waltham, MA USA; Universidade Federal do Rio De Janeiro COPPE/EE/IF, Rio de Janeiro, Brazil; Electrical Circuits Department, Federal University of Juiz de Fora (UFJF), Juiz de Fora, Brazil; Federal University of Sao Joao del Rei (UFSJ), Sao Joao del Rei, Brazil; Instituto de Fisica, Universidade de Sao Paulo, São Paulo, Brazil; Physics Department, Brookhaven National Laboratory, Upton, NY USA; National Institute of Physics and Nuclear Engineering, Bucharest, Romania; Physics Department, National Institute for Research and Development of Isotopic and Molecular Technologies, Cluj Napoca, Romania; University Politehnica Bucharest, Bucharest, Romania; West University in Timisoara, Timisoara, Romania; Departamento de Física, Universidad de Buenos Aires, Buenos Aires, Argentina; Cavendish Laboratory, University of Cambridge, Cambridge, UK; Department of Physics, Carleton University, Ottawa, ON Canada; CERN, Geneva, Switzerland; Enrico Fermi Institute, University of Chicago, Chicago, IL USA; Departamento de Física, Pontificia Universidad Católica de Chile, Santiago, Chile; Departamento de Física, Universidad Técnica Federico Santa María, Valparaiso, Chile; Institute of High Energy Physics, Chinese Academy of Sciences, Beijing, China; Department of Modern Physics, University of Science and Technology of China, Hefei, Anhui China; Department of Physics, Nanjing University, Nanjing, Jiangsu China; School of Physics, Shandong University, Shandong, China; Shanghai Key Laboratory for Particle Physics and Cosmology, Department of Physics and Astronomy, Shanghai Jiao Tong University, Shanghai, China; Physics Department, Tsinghua University, Beijing, 100084 China; Laboratoire de Physique Corpusculaire, Clermont Université and Université Blaise Pascal and CNRS/IN2P3, Clermont-Ferrand, France; Nevis Laboratory, Columbia University, Irvington, NY USA; Niels Bohr Institute, University of Copenhagen, Copenhagen, Denmark; INFN Gruppo Collegato di Cosenza, Laboratori Nazionali di Frascati, Frascati, Italy; Dipartimento di Fisica, Università della Calabria, Rende, Italy; AGH University of Science and Technology, Faculty of Physics and Applied Computer Science, Kraków, Poland; Marian Smoluchowski Institute of Physics, Jagiellonian University, Kraków, Poland; Institute of Nuclear Physics, Polish Academy of Sciences, Kraków, Poland; Physics Department, Southern Methodist University, Dallas, TX USA; Physics Department, University of Texas at Dallas, Richardson, TX USA; DESY, Hamburg and Zeuthen, Germany; Institut für Experimentelle Physik IV, Technische Universität Dortmund, Dortmund, Germany; Institut für Kern- und Teilchenphysik, Technische Universität Dresden, Dresden, Germany; Department of Physics, Duke University, Durham, NC USA; SUPA-School of Physics and Astronomy, University of Edinburgh, Edinburgh, UK; INFN Laboratori Nazionali di Frascati, Frascati, Italy; Fakultät für Mathematik und Physik, Albert-Ludwigs-Universität, Freiburg, Germany; Section de Physique, Université de Genève, Geneva, Switzerland; INFN Sezione di Genova, Genoa, Italy; Dipartimento di Fisica, Università di Genova, Genoa, Italy; E. Andronikashvili Institute of Physics, Iv. Javakhishvili Tbilisi State University, Tbilisi, Georgia; High Energy Physics Institute, Tbilisi State University, Tbilisi, Georgia; II Physikalisches Institut, Justus-Liebig-Universität Giessen, Giessen, Germany; SUPA-School of Physics and Astronomy, University of Glasgow, Glasgow, UK; II Physikalisches Institut, Georg-August-Universität, Göttingen, Germany; Laboratoire de Physique Subatomique et de Cosmologie, Université Grenoble-Alpes, CNRS/IN2P3, Grenoble, France; Department of Physics, Hampton University, Hampton, VA USA; Laboratory for Particle Physics and Cosmology, Harvard University, Cambridge, MA USA; Kirchhoff-Institut für Physik, Ruprecht-Karls-Universität Heidelberg, Heidelberg, Germany; Physikalisches Institut, Ruprecht-Karls-Universität Heidelberg, Heidelberg, Germany; ZITI Institut für technische Informatik, Ruprecht-Karls-Universität Heidelberg, Mannheim, Germany; Faculty of Applied Information Science, Hiroshima Institute of Technology, Hiroshima, Japan; Department of Physics, The Chinese University of Hong Kong, Shatin, N.T. Hong Kong; Department of Physics, The University of Hong Kong, Hong Kong, China; Department of Physics, The Hong Kong University of Science and Technology, Clear Water Bay, Kowloon, Hong Kong China; Department of Physics, Indiana University, Bloomington, IN USA; Institut für Astro- und Teilchenphysik, Leopold-Franzens-Universität, Innsbruck, Austria; University of Iowa, Iowa City, IA USA; Department of Physics and Astronomy, Iowa State University, Ames, IA USA; Joint Institute for Nuclear Research, JINR Dubna, Dubna, Russia; KEK, High Energy Accelerator Research Organization, Tsukuba, Japan; Graduate School of Science, Kobe University, Kobe, Japan; Faculty of Science, Kyoto University, Kyoto, Japan; Kyoto University of Education, Kyoto, Japan; Department of Physics, Kyushu University, Fukuoka, Japan; Instituto de Física La Plata, Universidad Nacional de La Plata and CONICET, La Plata, Argentina; Physics Department, Lancaster University, Lancaster, UK; INFN Sezione di Lecce, Lecce, Italy; Dipartimento di Matematica e Fisica, Università del Salento, Lecce, Italy; Oliver Lodge Laboratory, University of Liverpool, Liverpool, UK; Department of Physics, Jožef Stefan Institute and University of Ljubljana, Ljubljana, Slovenia; School of Physics and Astronomy, Queen Mary University of London, London, UK; Department of Physics, Royal Holloway University of London, Surrey, UK; Department of Physics and Astronomy, University College London, London, UK; Louisiana Tech University, Ruston, LA USA; Laboratoire de Physique Nucléaire et de Hautes Energies, UPMC and Université Paris-Diderot and CNRS/IN2P3, Paris, France; Fysiska institutionen, Lunds universitet, Lund, Sweden; Departamento de Fisica Teorica C-15, Universidad Autonoma de Madrid, Madrid, Spain; Institut für Physik, Universität Mainz, Mainz, Germany; School of Physics and Astronomy, University of Manchester, Manchester, UK; CPPM, Aix-Marseille Université and CNRS/IN2P3, Marseille, France; Department of Physics, University of Massachusetts, Amherst, MA USA; Department of Physics, McGill University, Montreal, QC Canada; School of Physics, University of Melbourne, Melbourne, VIC Australia; Department of Physics, The University of Michigan, Ann Arbor, MI USA; Department of Physics and Astronomy, Michigan State University, East Lansing, MI USA; INFN Sezione di Milano, Milan, Italy; Dipartimento di Fisica, Università di Milano, Milan, Italy; B.I. Stepanov Institute of Physics, National Academy of Sciences of Belarus, Minsk, Republic of Belarus; National Scientific and Educational Centre for Particle and High Energy Physics, Minsk, Republic of Belarus; Department of Physics, Massachusetts Institute of Technology, Cambridge, MA USA; Group of Particle Physics, University of Montreal, Montreal, QC Canada; P.N. Lebedev Institute of Physics, Academy of Sciences, Moscow, Russia; Institute for Theoretical and Experimental Physics (ITEP), Moscow, Russia; National Research Nuclear University MEPhI, Moscow, Russia; D.V. Skobeltsyn Institute of Nuclear Physics, M.V. Lomonosov Moscow State University, Moscow, Russia; Fakultät für Physik, Ludwig-Maximilians-Universität München, Munich, Germany; Max-Planck-Institut für Physik (Werner-Heisenberg-Institut), Munich, Germany; Nagasaki Institute of Applied Science, Nagasaki, Japan; Graduate School of Science and Kobayashi-Maskawa Institute, Nagoya University, Nagoya, Japan; INFN Sezione di Napoli, Naples, Italy; Dipartimento di Fisica, Università di Napoli, Naples, Italy; Department of Physics and Astronomy, University of New Mexico, Albuquerque, NM USA; Institute for Mathematics, Astrophysics and Particle Physics, Radboud University Nijmegen/Nikhef, Nijmegen, The Netherlands; Nikhef National Institute for Subatomic Physics and University of Amsterdam, Amsterdam, The Netherlands; Department of Physics, Northern Illinois University, De Kalb, IL USA; Budker Institute of Nuclear Physics, SB RAS, Novosibirsk, Russia; Department of Physics, New York University, New York, NY USA; Ohio State University, Columbus, OH USA; Faculty of Science, Okayama University, Okayama, Japan; Homer L. Dodge Department of Physics and Astronomy, University of Oklahoma, Norman, OK USA; Department of Physics, Oklahoma State University, Stillwater, OK USA; Palacký University, RCPTM, Olomouc, Czech Republic; Center for High Energy Physics, University of Oregon, Eugene, OR USA; LAL, Université Paris-Sud and CNRS/IN2P3, Orsay, France; Graduate School of Science, Osaka University, Osaka, Japan; Department of Physics, University of Oslo, Oslo, Norway; Department of Physics, Oxford University, Oxford, UK; INFN Sezione di Pavia, Pavia, Italy; Dipartimento di Fisica, Università di Pavia, Pavia, Italy; Department of Physics, University of Pennsylvania, Philadelphia, PA USA; National Research Centre “Kurchatov Institute” B.P.Konstantinov Petersburg Nuclear Physics Institute, St. Petersburg, Russia; INFN Sezione di Pisa, Pisa, Italy; Dipartimento di Fisica E. Fermi, Università di Pisa, Pisa, Italy; Department of Physics and Astronomy, University of Pittsburgh, Pittsburgh, PA USA; Laboratório de Instrumentaçãoe Física Experimental de Partículas-LIP, Lisbon, Portugal; Faculdade de Ciências, Universidade de Lisboa, Lisbon, Portugal; Department of Physics, University of Coimbra, Coimbra, Portugal; Centro de Física Nuclear da Universidade de Lisboa, Lisbon, Portugal; Departamento de Fisica, Universidade do Minho, Braga, Portugal; Departamento de Fisica Teorica y del Cosmos and CAFPE, Universidad de Granada, Granada, Spain; Dep Fisica and CEFITEC of Faculdade de Ciencias e Tecnologia, Universidade Nova de Lisboa, Caparica, Portugal; Institute of Physics, Academy of Sciences of the Czech Republic, Prague, Czech Republic; Czech Technical University in Prague, Prague, Czech Republic; Faculty of Mathematics and Physics, Charles University in Prague, Prague, Czech Republic; State Research Center Institute for High Energy Physics, Protvino, Russia; Particle Physics Department, Rutherford Appleton Laboratory, Didcot, UK; INFN Sezione di Roma, Rome, Italy; Dipartimento di Fisica, Sapienza Università di Roma, Rome, Italy; INFN Sezione di Roma Tor Vergata, Rome, Italy; Dipartimento di Fisica, Università di Roma Tor Vergata, Rome, Italy; INFN Sezione di Roma Tre, Rome, Italy; Dipartimento di Matematica e Fisica, Università Roma Tre, Rome, Italy; Faculté des Sciences Ain Chock, Réseau Universitaire de Physique des Hautes Energies-Université Hassan II, Casablanca, Morocco; Centre National de l’Energie des Sciences Techniques Nucleaires, Rabat, Morocco; Faculté des Sciences Semlalia, Université Cadi Ayyad, LPHEA-Marrakech, Marrakech, Morocco; Faculté des Sciences, Université Mohamed Premier and LPTPM, Oujda, Morocco; Faculté des Sciences, Université Mohammed V-Agdal, Rabat, Morocco; DSM/IRFU (Institut de Recherches sur les Lois Fondamentales de l’Univers), CEA Saclay (Commissariat à l’Energie Atomique et aux Energies Alternatives), Gif-sur-Yvette, France; Santa Cruz Institute for Particle Physics, University of California Santa Cruz, Santa Cruz, CA USA; Department of Physics, University of Washington, Seattle, WA USA; Department of Physics and Astronomy, University of Sheffield, Sheffield, UK; Department of Physics, Shinshu University, Nagano, Japan; Fachbereich Physik, Universität Siegen, Siegen, Germany; Department of Physics, Simon Fraser University, Burnaby, BC Canada; SLAC National Accelerator Laboratory, Stanford, CA USA; Faculty of Mathematics, Physics and Informatics, Comenius University, Bratislava, Slovak Republic; Department of Subnuclear Physics, Institute of Experimental Physics of the Slovak Academy of Sciences, Kosice, Slovak Republic; Department of Physics, University of Cape Town, Cape Town, South Africa; Department of Physics, University of Johannesburg, Johannesburg, South Africa; School of Physics, University of the Witwatersrand, Johannesburg, South Africa; Department of Physics, Stockholm University, Stockholm, Sweden; The Oskar Klein Centre, Stockholm, Sweden; Physics Department, Royal Institute of Technology, Stockholm, Sweden; Departments of Physics and Astronomy and Chemistry, Stony Brook University, Stony Brook, NY USA; Department of Physics and Astronomy, University of Sussex, Brighton, UK; School of Physics, University of Sydney, Sydney, Australia; Institute of Physics, Academia Sinica, Taipei, Taiwan; Department of Physics, Technion: Israel Institute of Technology, Haifa, Israel; Raymond and Beverly Sackler School of Physics and Astronomy, Tel Aviv University, Tel Aviv, Israel; Department of Physics, Aristotle University of Thessaloniki, Thessaloníki, Greece; International Center for Elementary Particle Physics and Department of Physics, The University of Tokyo, Tokyo, Japan; Graduate School of Science and Technology, Tokyo Metropolitan University, Tokyo, Japan; Department of Physics, Tokyo Institute of Technology, Tokyo, Japan; Department of Physics, University of Toronto, Toronto, ON Canada; TRIUMF, Vancouver, BC Canada; Department of Physics and Astronomy, York University, Toronto, ON Canada; Faculty of Pure and Applied Sciences, University of Tsukuba, Tsukuba, Japan; Department of Physics and Astronomy, Tufts University, Medford, MA USA; Centro de Investigaciones, Universidad Antonio Narino, Bogotá, Colombia; Department of Physics and Astronomy, University of California Irvine, Irvine, CA USA; INFN Gruppo Collegato di Udine, Sezione di Trieste, Udine, Italy; ICTP, Trieste, Italy; Dipartimento di Chimica, Fisica e Ambiente, Università di Udine, Udine, Italy; Department of Physics, University of Illinois, Urbana, IL USA; Department of Physics and Astronomy, University of Uppsala, Uppsala, Sweden; Instituto de Física Corpuscular (IFIC) and Departamento de Física Atómica, Molecular y Nuclear and Departamento de Ingeniería Electrónica and Instituto de Microelectrónica de Barcelona (IMB-CNM), University of Valencia and CSIC, Valencia, Spain; Department of Physics, University of British Columbia, Vancouver, BC Canada; Department of Physics and Astronomy, University of Victoria, Victoria, BC Canada; Department of Physics, University of Warwick, Coventry, UK; Waseda University, Tokyo, Japan; Department of Particle Physics, The Weizmann Institute of Science, Rehovot, Israel; Department of Physics, University of Wisconsin, Madison, WI USA; Fakultät für Physik und Astronomie, Julius-Maximilians-Universität, Würzburg, Germany; Fachbereich C Physik, Bergische Universität Wuppertal, Wuppertal, Germany; Department of Physics, Yale University, New Haven, CT USA; Yerevan Physics Institute, Yerevan, Armenia; Centre de Calcul de l’Institut National de Physique Nucléaire et de Physique des Particules (IN2P3), Villeurbanne, France; CERN, 1211 Geneva 23, Switzerland

## Abstract

Searches for pair-produced scalar leptoquarks are performed using 20 fb$^{-1}$ of proton–proton collision data provided by the LHC and recorded by the ATLAS detector at $\sqrt{s}=8$ TeV. Events with two electrons (muons) and two or more jets in the final state are used to search for first (second)-generation leptoquarks. The results from two previously published ATLAS analyses are interpreted in terms of third-generation leptoquarks decaying to $b\nu _{\tau }\bar{b}\bar{\nu _{\tau }}$ and $t\nu _{\tau }\bar{t}\bar{\nu _{\tau }}$ final states. No statistically significant excess above the Standard Model expectation is observed in any channel and scalar leptoquarks are excluded at 95 % CL with masses up to $m_{\mathrm {LQ1}}<$ 1050 GeV for first-generation leptoquarks, $m_{\mathrm {LQ2}}<$ 1000 GeV for second-generation leptoquarks, $m_{\mathrm {LQ3}}<$ 625 GeV for third-generation leptoquarks in the $b\nu _{\tau }\bar{b}\bar{\nu _{\tau }}$ channel, and 200 $< m_{\mathrm {LQ3}}<$ 640 GeV in the $t\nu _{\tau }\bar{t}\bar{\nu _{\tau }}$ channel.

## Introduction

Leptoquarks (LQ) are predicted by many extensions of the Standard Model (SM) [1–7] and may provide an explanation for the many observed similarities between the quark and lepton sectors of the SM. LQs are colour-triplet bosons with fractional electric charge. They carry non-zero values of both baryon and lepton number [8]. They can be either scalar or vector bosons and are expected to decay directly to lepton–quark pairs (where the lepton can be either charged or neutral).

The coupling strength between scalar LQs and the lepton-quark pairs depends on a single Yukawa coupling, termed $\lambda _{\mathrm {LQ} \rightarrow \ell q}$. The additional magnetic moment and electric quadrupole moment interactions of vector LQs are governed by two coupling constants [9]. The coupling constants for both the scalar and vector LQs, and the branching fraction of the LQ decay into a quark and a charged lepton, $\beta$, are model dependent. The production cross-section and couplings of vector LQs are enhanced relative to the contribution of scalar LQs, although the acceptance is expected to be similar in both cases. This analysis considers the simpler scenario of scalar LQ pair-production, for which the form of the interaction is known and which provides more conservative limits on LQ pair-production than for vector LQ pair-production.

In proton–proton collisions, LQs can be produced singly and in pairs. The production of single LQs, which happens at hadron colliders in association with a lepton, depends directly on the unknown Yukawa coupling $\lambda _{\mathrm {LQ} \rightarrow \ell q}$. However, LQ pair-production is not sensitive to the value of the coupling. In *pp* collisions with a centre-of-mass energy $\sqrt{s}$ $=$ 8 TeV, the dominant pair-production mechanism for LQ masses below $\sim$1 TeV is gluon fusion, while the *qq*-annihilation production process becomes increasingly important with increasing LQ mass.

The minimal Buchmüller–Rückl–Wyler model (mBRW) [10] is used as a benchmark model for scalar LQ production. It postulates additional constraints on the LQ properties, namely that the couplings have to be purely chiral, and makes the assumption that LQs are grouped into three families (first, second and third-generation) that couple only to leptons and quarks within the same generation. The latter requirement excludes the possibility of flavour-changing neutral currents (FCNC) [11], which have not been observed to date.

Previous searches for pair-produced LQs have been performed by the ATLAS Collaboration with 1.03 fb$^{-1}$ of data collected at $\sqrt{s}$ $=$ 7 TeV, excluding at 95 % confidence level (CL) the existence of scalar LQs with masses below 660 (607) GeV for first-generation LQs at $\beta =1$ (0.5) [12] and 685 (594) GeV for second-generation LQs at $\beta =1$ (0.5) [13]. The CMS Collaboration excluded at 95 % CL the existence of scalar LQs with masses below 830 (640) GeV for first-generation LQs at $\beta =1$ (0.5) and 840 (650) GeV for second-generation LQs at $\beta =1$ (0.5) with 5.0 fb$^{-1}$ of data collected at $\sqrt{s}$ $=$ 7 TeV [14].

Pair-produced third-generation scalar LQs decaying to $b\nu _{\tau }\bar{b}\bar{\nu _{\tau }}$ have been excluded by the CMS Collaboration for masses below 700 GeV at $\beta = 0$, and for masses below 560 GeV  over the full $\beta$ range using 19.7 fb$^{-1}$ of data collected at $\sqrt{s}$ $=$ 8 TeV [15]. Third-generation scalar LQs have been excluded in the $b\tau ^{+}\bar{b}\tau ^{-}$ channel at $\beta = 1$ for masses up to 740 GeV by the CMS Collaboration using 19.7 fb$^{-1}$ of data collected at $\sqrt{s}$ $=$ 8 TeV [16], and by the ATLAS Collaboration at $\beta = 1$ for masses up to 534 GeV using 4.7 fb$^{-1}$ of data collected at $\sqrt{s}$ $=$ 7 TeV [17]. The CMS Collaboration also excluded third-generation scalar LQs in the $t\tau ^{-}\bar{t}\tau ^{+}$ channel at $\beta = 1$ for masses up to 685 GeV  using 19.7 fb$^{-1}$ of data collected at $\sqrt{s}$ $=$ 8 TeV [15].

In this paper, searches for pair-produced first- and second-generation scalar LQs ($\mathrm {LQ}$1 and $\mathrm {LQ}$2, respectively) are performed by selecting events with two electrons or muons plus two jets in the final state (denoted by *eejj*  and $\mu \mu$*jj*, respectively). In addition, limits are placed on pair-produced third-generation scalar LQs ($\mathrm {LQ}$3) by reinterpreting ATLAS searches for supersymmetry (SUSY) in two different channels [18, 19]. LQ production and decay mechanisms can be similar to those of stop quarks ($\tilde{t}$) and sbottom quarks ($\tilde{b}$). For example, $\tilde{t}\tilde{t}\rightarrow tt \tilde{\chi }^{0} \tilde{\chi }^{0}$ gives the same event topology as $\mathrm {LQ}3\,\overline{\mathrm {LQ}3}\rightarrow$ $t\nu _{\tau }\bar{t}\bar{\nu _{\tau }}$ in the limit where the neutralino ($\tilde{\chi }^{0}$) is massless. Two ATLAS analyses optimised for these SUSY processes are therefore used to set limits on the equivalent LQ decay processes: $\mathrm {LQ}3\,\overline{\mathrm {LQ}3}\rightarrow$ $b\nu _{\tau }\bar{b}\bar{\nu _{\tau }}$ and $\mathrm {LQ}3\,\overline{\mathrm {LQ}3}\rightarrow$ $t\nu _{\tau }\bar{t}\bar{\nu _{\tau }}$.

The results for each $\mathrm {LQ}$3 channel cannot be combined since the parent LQs have different electric charges in the two cases ($-\frac{1}{3}e$ for the $\mathrm {LQ}3\,\overline{\mathrm {LQ}3}\rightarrow$ $b\nu _{\tau }\bar{b}\bar{\nu _{\tau }}$ channel and $\frac{2}{3}e$ for the $\mathrm {LQ}3\,\overline{\mathrm {LQ}3}\rightarrow$ $t\nu _{\tau }\bar{t}\bar{\nu _{\tau }}$ channel, where *e* is the elementary electric charge). The branching fractions of $\mathrm {LQ}$3 decays to $b\nu _{\tau }$ and $t\nu _{\tau }$ are assumed to be equal to 100 % in each case. Although complementary decays of a charge $-\frac{1}{3}e$ ($\frac{2}{3}e$) LQ into a $t\tau ^{-}\bar{t}\tau ^{+}$ ($b\tau ^{+}\bar{b}\tau ^{-}$) final state are also allowed, kinematic suppression factors which favour LQ decays to *b*-quarks over *t*-quarks and the relative strengths of the Yukawa couplings would have to be considered. Since these suppression factors are model dependent, limits are not provided as a function of $\beta$ for the $\mathrm {LQ}$3 channels.

## The ATLAS detector

The ATLAS experiment [20] is a multi-purpose detector with a forward–backward symmetric cylindrical geometry and nearly 4$\pi$ coverage in solid angle. The three major sub-components of ATLAS are the tracking detector, the calorimeter and the muon spectrometer. Charged-particle tracks and vertices are reconstructed by the inner detector (ID) tracking system, comprising silicon pixel and microstrip detectors covering the pseudorapidity^1^ range $|\eta |$ $<$ 2.5, and a straw tube tracker that covers $|\eta |$ $<$ 2.0. The ID is immersed in a homogeneous 2 T magnetic field provided by a solenoid. Electron, photon, jet and tau energies are measured with sampling calorimeters. The ATLAS calorimeter system covers a pseudorapidity range of $|\eta |$ $<$ 4.9. Within the region $|\eta |$ $<$ 3.2, electromagnetic calorimetry is provided by barrel and endcap high-granularity lead/liquid argon (LAr) calorimeters, with an additional thin LAr presampler covering $|\eta |$ $<$ 1.8, to correct for energy loss in material upstream of the calorimeters. Hadronic calorimetry is provided by a steel/scintillator-tile calorimeter, segmented into three barrel structures within $|\eta |$ $<$ 1.7, and two copper/LAr hadronic endcap calorimeters. The forward region (3.1 $<$ $|\eta |$ $<$ 4.9) is instrumented by a LAr calorimeter with copper (electromagnetic) and tungsten (hadronic) absorbers. Surrounding the calorimeters is a muon spectrometer (MS) with air-core toroids, a system of precision tracking chambers providing coverage over $|\eta |$ $<$ 2.7, and detectors with triggering capabilities over $|\eta |$ $<$ 2.4 to provide precise muon identification and momentum measurements.

## Data and Monte Carlo samples

The results presented here are based on proton–proton collision data at a centre-of-mass energy of $\sqrt{s}$ $=$ 8 TeV, collected by the ATLAS detector at the LHC during 2012. Data samples corresponding to an integrated luminosity of 20.3fb$^{-1}$ are used by all channels except for the $\mathrm {LQ}3\,\overline{\mathrm {LQ}3}\rightarrow b\nu _{\tau }\bar{b}\bar{\nu _{\tau }}$ analysis which uses 20.1 fb$^{-1}$ because of requirements made by the trigger used in the analysis.

Simulated signal events of pair-produced scalar LQs decaying to $e^{+}e^{-}q\bar{q}$, $\mu ^{+}\mu ^{-}q\bar{q}$, $t\nu _{\tau }\bar{t}\bar{\nu _{\tau }}$, and $b\nu _{\tau }\bar{b}\bar{\nu _{\tau }}$ final states are produced using the Pythia 8.160 [21] event generator with CTEQ6L1 [22] parton distribution functions (PDFs). The coupling $\lambda _{\mathrm {LQ} \rightarrow \ell q}$ which determines the LQ lifetime and width [23] is set to $\sqrt{0.01\times 4\pi \alpha }$, where $\alpha$ is the fine-structure constant. This value gives the LQ a full width of less than 100 MeV, which is much smaller than the detector resolution. For LQ masses in the ranges considered here (200 GeV $\le$ $m_{\mathrm {LQ}}$ $\le$ 1300 GeV, in steps of 50 GeV), the value of the coupling used is such that the LQs can be considered to decay promptly. The production cross-section of pair-produced LQs is assumed to be independent of the coupling strength. The signal process is normalised to the expected next-to-leading-order (NLO) cross-sections for scalar LQ pair-production [24]. The signal production cross-section is 23.5 fb for a LQ mass of 600 GeV, and 0.40 fb for a 1 TeV LQ and is the same for each generation.

### Monte Carlo for background predictions

The Monte Carlo (MC) samples used to estimate the contributions from SM backgrounds to the $\mathrm {LQ}$1 and $\mathrm {LQ}$2 searches are discussed here. Details about the MC models used for estimating backgrounds in the $\mathrm {LQ}$3 searches are available in Refs. [18] (for the $b\nu _{\tau }\bar{b}\bar{\nu _{\tau }}$ channel) and [19] (for the $t\nu _{\tau }\bar{t}\bar{\nu _{\tau }}$ channel).

The MC samples used to model the $$Z/\gamma ^*$$$$+$$jets background with a dilepton invariant mass ($$m_{\mathrm {\ell \ell }} $$) less than 120 GeV and high-mass Drell–Yan backgrounds ($$m_{\mathrm {\ell \ell }} \ge $$  120 GeV) are generated with SHERPA 1.4.1 [25]. The high-mass Drell–Yan samples are generated assuming massive *c*- and *b*-quarks instead of the conventional massless treatment.

Samples of $$t\bar{t}$$ events are produced with POWHEG box [26, 27] interfaced with PYTHIA 6. MC samples representing the *WW*, *WZ*, and *ZZ* diboson decays are generated with HERWIG 6.52 [28] and use the AUET2 [29] values for the tunable parameters (the ‘AUET2 MC tune’). Samples of single-top-quark events in the *Wt* and *s*-channel are generated with MC@NLO 4.01 [30, 31] and the AUET2 MC tune, while the *t*-channel samples are generated with AcerMC 3.8 [32] interfaced with PYTHIA 8 and use the AUET2B [33] MC tune. The hadronisation and parton showering of the samples produced with MC@NLO are done using HERWIG 6.52 coupled to JIMMY 4.31 [34]. The $$W$$$$+$$jets samples are produced with ALPGEN 2.14 interfaced with JIMMY 4.31, also with the AUET2 MC tune applied. The choice of PDFs used to produce the MC simulated samples is generator dependent: AcerMC, PYTHIA, HERWIG and ALPGEN use CTEQ6L1, while MC@NLO uses CT10 [35]. For all samples, the detector response is modelled [36] using GEANT4 [37], except for the Drell–Yan background samples, which use a fast detector simulation where the calorimeter response is parameterised. The differences between fast and full simulation in terms of kinematic spectra and modelling of relevant objects are evaluated to be negligible.

The cross-sections of background processes used in the analysis are taken from theoretical predictions. Single-top production cross-sections in the *s*-channel [38], *t*-channel [39], and in associated production with a *W* boson [40], are calculated to NLO$$+$$NNLL accuracy. $$W$$$$+$$jets and $$Z\rightarrow \tau \tau $$ cross-sections with NNLO accuracy are used [41]. The cross-sections for *WW*, *WZ*, and *ZZ* processes are calculated at NLO [42, 43]. The theoretical cross-section for *WW* production is scaled by a factor 1.2 and the uncertainty is increased by an extra 20 %, in order to take into account the ATLAS [44] and CMS measurements [45], which showed an excess in data at the level of 20 % (see Refs. [46, 47] for more discussion about possible causes of the excess).

For the $$Z/\gamma ^*$$$$+$$jets and $$t\bar{t}$$ backgrounds, LO and NLO cross-sections, respectively are used. These backgrounds are constrained using two control regions (CRs), as described in Sect. 4.5.

## Searches for first- and second-generation LQs

The first- and second-generation analyses exploit similarities in the final states and use common search strategies to select dilepton plus dijet final states. Control regions are used to constrain estimates of the dominant backgrounds to the data. A set of discriminating variables is used to define signal regions (SRs) that are used for a counting analysis.

### Trigger and data collection

Selected data events are required to have all relevant components of the ATLAS detector in good working condition. For the $\mathrm {LQ}$1 (*eejj*) analysis, the trigger requires at least two electromagnetic calorimeter clusters, defined as energy deposits in the cells of the electromagnetic calorimeter. The leading cluster is required to have transverse momentum $p_{\mathrm {T}}$ > 35 GeV and the sub-leading one $p_{\mathrm {T}}$ > 25 GeV. This trigger selects electrons without imposing any requirement on the isolation and this allows a data-driven estimate of the background contribution from jets in the final state that pass the electron selection, as described in detail in Ref. [48]. The trigger is 98 % efficient with respect to the offline selection, which requires $p_{\mathrm {T}}$ above 40 (30) GeV for the leading (sub-leading) electron.

For the $\mathrm {LQ}$2 ($\mu \mu$*jj*) analysis, events are selected from data using a trigger which requires the presence of at least one muon candidate in the event with $p_{\mathrm {T}}$ above 36 GeV. This trigger is fully efficient relative to the offline selection for muons with $p_{\mathrm {T}}$ above 40 GeV [49].

### Object selection

Electrons are selected and identified by imposing requirements on the shape of the cluster of energy deposits in the calorimeter, as well as on the quality of the track, and on the track-to-cluster matching. The identification efficiency is on average 85 % [50]. Electron candidates must have transverse energy $E_{\mathrm {T}}>30$ GeV and $|\eta |<2.47$. Electron candidates associated with clusters in the transition region between the barrel and endcap calorimeters (1.37 < $|\eta |$ < 1.52) are excluded. All electrons are required to be reconstructed with cluster-based or combined cluster- and track-based algorithms and to satisfy calorimeter quality criteria. Requirements are made on the transverse ($|d_0|$) and longitudinal ($|z_0|$) impact parameters of the electron relative to the primary vertex and must satisfy $|d_0| < 1$ mm and $|z_0| < 5$ mm. In addition, electrons are required to be isolated by imposing requirements on the $E_{\mathrm {T}}^{\mathrm {\Delta R<0.2}}$ measured in the calorimeter within a cone of size $\Delta R=\sqrt{(\Delta \eta )^2 + (\Delta \phi )^2} = 0.2$ around the electron cluster excluding the electron cluster energy, and corrected to account for leakage (i.e. energy deposited by the electron outside of the cluster) and the average number of proton–proton interactions per bunch-crossing. The isolation requirements are optimised for high-$p_{\mathrm {T}}$ electrons following the strategy in Ref. [48]. The leading electron is required to have $E_{\mathrm {T}}^{\mathrm {\Delta R<0.2}}<0.007\times E_{\mathrm {T}}+5$ GeV, and the sub-leading electron is required to have $E_{\mathrm {T}}^{\mathrm {\Delta R<0.2}}<0.022\times E_{\mathrm {T}}+6$ GeV.

Muon tracks are reconstructed independently in the ID and the MS. Tracks are required to have a minimum number of hits in each system, and must be compatible in terms of geometrical and momentum matching. In particular, in order to prevent mis-measurements at high $p_{\mathrm {T}}$, muons are required to have hits in all three MS stations, as described in Ref. [48]. In order to increase the muon identification efficiency, when one muon in the event satisfies the three-stations requirement, the criteria for the second muon in the event are relaxed to require hits in only two MS stations. Information from both the ID and MS is used in a combined fit to refine the measurement of the momentum of each muon [51]. Muon candidates are required to have $p_{\mathrm {T}}>40$ GeV, $|\eta |$ < 2.4, $|d_0|<0.2$ mm and $|z_0|<1.0$ mm. Muons must also pass a relative-isolation requirement $p_{\mathrm {T}}^{\mathrm {\Delta R<0.2}}/p_{\mathrm {T}}<0.2$, where $p_{\mathrm {T}}^{\mathrm {\Delta R<0.2}}$ is the sum of the transverse momenta of all the tracks with $p_{\mathrm {T}}$ above 1 GeV (except for the muon track) within a cone of $\Delta R$ < 0.2 around the muon track, and $p_{\mathrm {T}}$ is the transverse momentum of the muon.

Jets are reconstructed from clusters of energy deposits detected in the calorimeter using the anti-$k_{t}$ algorithm [52] with a radius parameter $R=0.4$ [53]. They are calibrated using energy- and $\eta$-dependent correction factors derived from simulation and with residual corrections from in-situ measurements. The jets used in the analysis must satisfy $p_{\mathrm {T}}$ > 30 GeV and $|\eta |$ < 2.8. Jets reconstructed within a cone of $\Delta R=0.4$ around a selected electron or muon are removed. Additional jet quality criteria are also applied to remove fake jets caused by detector effects. A detailed description of the jet energy scale measurement and its systematic uncertainties is given in Ref. [54].

### Event pre-selection

Multiple *pp* interactions during bunch-crossings (pile-up) can give rise to multiple reconstructed vertices in events. The primary vertex of the event, from which the leptons are required to originate, is defined as the one with the largest sum of squared transverse momenta of its associated tracks. Events are selected if they contain a primary vertex with at least three associated tracks satisfying $p_{\mathrm {T,track}} >$ 0.4 GeV.

MC events are corrected to better describe the data by applying a per-event weight to match the distribution of the average number of primary vertices observed in data. A weighting factor is also applied in order to improve the modelling of the vertex position in *z*. Scale factors are applied to account for differences in lepton identification and selection efficiency between data and MC simulation. The scale factors depend on the lepton kinematics and are described in detail in Ref. [51] for muons, and in Ref. [55] for electrons. The energy and momentum of the selected physics objects are corrected to account for the resolution and scale measured in data, as described in Ref. [51] for muons, in Ref. [55] for electrons and in Ref. [54] for jets.

Events are selected in the *eejj*  channel if they contain exactly two electrons with $p_{\mathrm {T}}$ > 40 (30) GeV for the leading (sub-leading) electron and at least two jets with $p_{\mathrm {T}}$ > 30 GeV. For the $\mu \mu$*jj*  channel, events are selected if they contain exactly two muons with $p_{\mathrm {T}}$ $>$ 40 GeV and opposite-sign charge, and at least two jets with $p_{\mathrm {T}}$ $>$ 30 GeV. No requirements are placed on the charges of the electron candidates due to inefficiencies in determining the charge of high-$p_{\mathrm {T}}$ tracks associated with electrons. These sets of requirements form the basic event ‘pre-selection’ for the analyses, which is used to build the control and signal regions discussed in the following sections.

### Signal regions

After applying the event pre-selection requirements, a set of signal regions is defined using additional kinematic variables in order to discriminate LQ signals from SM background processes and to enhance the signal-to-background ratio. The variables used are:

- $m_{\mathrm {\ell \ell }}$: The dilepton invariant mass.
- $S_{\mathrm {T}}$: The scalar sum of the transverse momenta of the two leading leptons and the two leading jets.
- $m_{\mathrm {LQ}}^{\mathrm {min}}$: The lowest reconstructed LQ mass in the event. The reconstructed masses of the two LQ candidates in the event ($m_{\mathrm {LQ}}^{\mathrm {min}}$ and $m_{\mathrm {LQ}}^{\mathrm {max}}$) are defined as the invariant masses of the two lepton–jet pairs with the smallest difference (and $m_{\mathrm {LQ}}^{\mathrm {min}}< m_{\mathrm {LQ}}^{\mathrm {max}}$).

Signal regions are determined by optimising the statistical significance as defined in Ref. [56]. The optimisation procedure is performed in a three-dimensional space constructed by $m_{\mathrm {\ell \ell }}$, $S_{\mathrm {T}}$ and $m_{\mathrm {LQ}}^{\mathrm {min}}$  for each of the signal mass points. Several adjacent mass points may be grouped into a single SR. The signal acceptance of the selection requirements is estimated to be $\approx$50 % in the $\mu \mu$*jj*  channel and between 65 and 80 % in the *eejj*  channel (assuming $\beta = 1.0$). The difference is due to tighter quality selection requirements in the $\mu \mu$*jj*  channel used to prevent muon mis-measurements in MS regions with poor alignment or missing chambers. The optimised signal regions are presented in Table 1 together with the mass of the corresponding LQ hypothesis. Each LQ mass hypothesis is tested in only one signal region, where limits on $\sigma \times \beta$ are extracted.

**Table 1:** The minimum values of $m_{\mathrm {\ell \ell }}$, $S_{\mathrm {T}}$, and $m_{\mathrm {LQ}}^{\mathrm {min}}$ used to define each of the signal regions targeting different LQ masses in the *eejj* and $\mu \mu$ *jj* channels. Each signal region is valid for one or more mass hypotheses, as shown in the second column

	LQ masses ($\mathrm{GeV}$)	$m_{\mathrm {\ell \ell }}$ ($\mathrm{GeV}$)	$S_{\mathrm {T}}$ ($\mathrm{GeV}$)	$m_{\mathrm {LQ}}^{\mathrm {min}}$ ($\mathrm{GeV}$)
SR1	300	130	460	210
SR2	350	160	550	250
SR3	400	160	590	280
SR4	450	160	670	370
SR5	500–550	180	760	410
SR6	600–650	180	850	490
SR7	700–750	180	950	580
SR8	800–1300	180	1190	610

### Background estimation

The main SM background processes to the $$\mathrm {LQ}$$1 and $$\mathrm {LQ}$$2 searches are the production of $$Z/\gamma ^*$$$$+$$jets events, $$t\bar{t}$$ events where both top quarks decay leptonically, and diboson events. Additional small contributions are expected from $$Z\rightarrow \tau \tau $$ and single-top processes. Multi-jets, $$W$$$$+$$jets, $$t\bar{t}$$ (where one or more top quarks decays hadronically), and single-top events with mis-identified or non-prompt leptons arising from hadron decays or photon conversions can also contribute. These fake lepton backgrounds are estimated separately in the *eejj*  and $$\mu \mu $$*jj*  channels using the same data-driven techniques as described in Ref. [48] and are found to be negligible for the $$\mu \mu $$*jj*  channel. Normalisation factors, derived using background-enriched control regions, are applied to the MC predictions for $$Z/\gamma ^*$$$$+$$jets and $$t\bar{t}$$ backgrounds to predict as accurately as possible the background in the signal regions. These control regions are constructed to be mutually exclusive to the signal region and the assumption is made that normalisation factors and their associated uncertainties in the signal region are the same as in the background-enriched control regions.

**Fig. 1:**
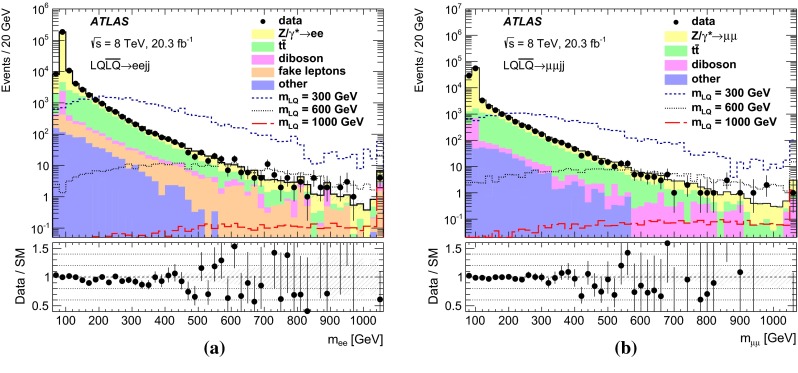
Distributions of the dilepton invariant mass ($m_{\mathrm {\ell \ell }}$) in the *eejj*  (*left*) and $\mu \mu$ *jj*  (*right*) channels after applying the pre-selection cuts. The signal model assumes $\beta = 1.0$. The last bin includes overflows. The ratio of the number of data events to the number of background events (and its statistical uncertainty) is also shown. The *hashed bands* represent all sources of statistical and systematic uncertainty on the background prediction

#### Control regions for $$Z/\gamma ^*$$$$+$$jets and $$t\bar{t}$$ backgrounds

Two control regions with negligible signal contributions are defined to validate the modelling accuracy of the MC simulated background events and to derive normalisation scale factors. The $$Z/\gamma ^*$$$$+$$jets control region is defined by the pre-selection requirements with an additional requirement of 60 $$<$$ $$m_{ee}$$ $$<$$ 120 GeV in the *eejj*  channel and 70 $$<$$ $$m_{\mu \mu }$$ $$<$$ 110 GeV in the $$\mu \mu $$*jj*  channel. These control regions define a pure sample of $$Z/\gamma ^*$$$$+$$jets events. The $$t\bar{t}$$ control region is defined in both channels by applying the pre-selection requirements, but demanding exactly one muon and one electron (both with $$p_{\mathrm {T}}$$ above 40 GeV) in the offline selection instead of two same-flavour leptons. In the case of the $$t\bar{t}$$ control region for the *eejj* channel, the trigger requirement is modified by requiring a single isolated electron with $$p_{\mathrm {T}}$$ above 24 GeV, which is fully efficient relative to the offline selection for electrons with $$p_{\mathrm {T}}$$ above 30 GeV. In both cases, the same selection criteria are applied to data and MC events.

Normalisation factors are applied to the MC predictions for the $$Z/\gamma ^*$$$$+$$jets and $$t\bar{t}$$ background processes. They are obtained by performing a combined maximum likelihood fit to the observed yields in the control regions and signal region under consideration. Systematic uncertainties on the predicted MC yields related to the uncertainty on the cross-sections are taken into account by the fit through the use of dedicated nuisance parameters. The fit procedure is performed using the HistFitter package [57], which is a tool based on the RooStats framework [58]. The normalisation scale factor obtained from a background-only fit for the $$Z/\gamma ^*$$$$+$$jets background in the *eejj*  ($$\mu \mu $$*jj*) channel is $$1.1\pm 0.2$$ ($$0.97\pm 0.15$$), while the normalisation scale factor for $$t\bar{t}$$ is $$1.10\pm 0.05$$ ($$1.01\pm 0.05$$). The fitted background scale factors have little sensitivity to the inclusion of signal regions and the eventual presence of a signal.

#### Kinematic distributions

The distributions of the kinematic variables after performing the background-only fits in the control regions, and applying the event pre-selection requirements are shown in Figs. , and for the data, background estimates, and for three LQ masses of 300, 600 and 1000 GeV (with $\beta = 1.0$).

**Fig. 2:**
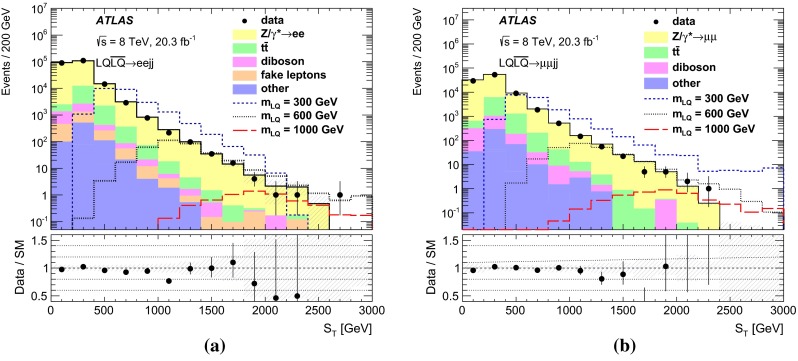
Distributions of the total scalar energy ($S_{\mathrm {T}}$) in the *eejj*  (*left*) and $\mu \mu$ *jj*  (*right*) channels after applying the pre-selection cuts. The signal model assumes $\beta = 1.0$. The last bin includes overflows. The ratio of the number of data events to the number of background events (and its statistical uncertainty) is also shown. The *hashed bands* represent all sources of statistical and systematic uncertainty on the background prediction

**Fig. 3:**
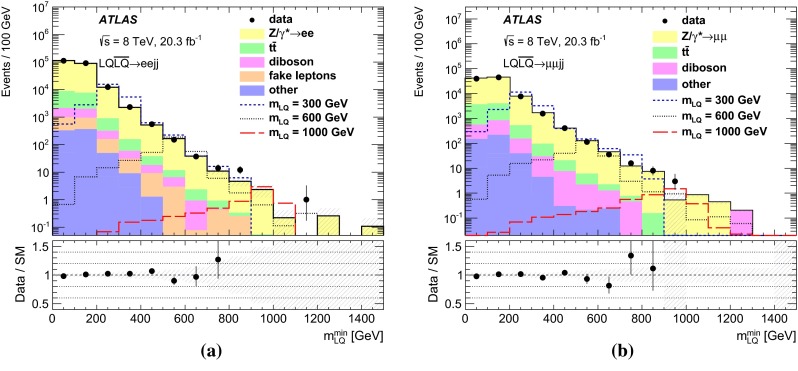
Distributions of the lowest reconstructed LQ mass ($m_{\mathrm {LQ}}^{\mathrm {min}}$) in the *eejj*  (*left*) and $\mu \mu$ *jj*  (*right*) channels after applying the pre-selection cuts. The signal model assumes $\beta = 1.0$. The last bin includes overflows. The ratio of the number of data events to the number of background events (and its statistical uncertainty) is also shown. The *hashed bands* represent all sources of statistical and systematic uncertainty on the background prediction

### Systematic uncertainties

The theoretical uncertainty on the NLO cross-section is taken into account for diboson, single-top, $$W$$$$+$$jets, and $$Z\rightarrow \tau \tau $$ processes. For the two dominant backgrounds ($$t\bar{t}$$ and $$Z/\gamma ^*$$$$+$$jets) the modelling uncertainties are estimated using the symmetrised deviation from unity of the ratio of data to MC events in the $$t\bar{t}$$ and $$Z/\gamma ^*$$$$+$$jets control regions, which is fitted with a linear function for $$S_{\mathrm {T}}>400$$ GeV. The modelling systematic uncertainty is then applied as a function of $$S_{\mathrm {T}}$$, in the form of a weighting factor. The choice of $$S_{\mathrm {T}}$$ for such a purpose is motivated by its sensitivity to mis-modelling of the kinematics of jets and leptons. It varies in the *eejj* ($$\mu \mu $$*jj*) channel between 8 % (10 %) and 25 % (30 %) for the $$Z/\gamma ^*$$$$+$$jets background and between 6 % (10 %) and 24 % (40 %) for the $$t\bar{t}$$ background. It increases for signal regions targeting higher $$m_{\mathrm {LQ}}$$.

**Table 2:** Background and signal yields in three representative signal regions for LQs with masses $m_{\mathrm {LQ}}=$ 300, 600 and 1000 GeV for the *eejj* channel (assuming $\beta$ = 1.0). The observed number of events is also shown. Statistical and systematic uncertainties are given

Yields	*eejj* channel		
	SR1	SR6	SR8
Observed	627	8	1
Total SM	$(6.4\pm 0.4)\times 10^2$	$11\pm 2$	$1.5\pm 0.4$
$Z/\gamma ^*\rightarrow \ell \ell$	$(3.2\pm 0.4)\times 10^2$	$7\pm 2$	$1.3\pm 0.4$
$Z\rightarrow \tau \tau$	$2.1\pm 0.3$	$<$0.01	$<$0.01
$t\bar{t}$	$(2.4\pm 0.2)\times 10^2$	$2.3\pm 0.5$	$0.12\pm 0.04$
Single top	$19\pm 3$	$<$0.01	$<$0.01
Diboson	$22\pm 3$	$0.8\pm 0.3$	$<$0.01
Fake leptons (including $W$ $+$jets)	$34\pm 6$	$0.410\pm 0.010$	$0.033\pm 0.006$
$m_{\mathrm {LQ}}$ $=$ 300 GeV	$(17.6\pm 0.9)\times 10^3$	–	–
$m_{\mathrm {LQ}}$ $=$ 600 GeV	–	$231\pm 13$	–
$m_{\mathrm {LQ}}$ $=$ 1000 GeV	–	–	$5.2\pm 0.3$

**Table 3:** Background and signal yields in three representative signal regions for LQs with masses $m_{\mathrm {LQ}}=$ 300, 600 and 1000 GeV for the $\mu \mu$ *jj* channel (assuming $\beta = 1.0$). The observed number of events is also shown. Statistical and systematic uncertainties are given

Yields	$\mu \mu$ *jj* channel		
	SR1	SR6	SR8
Observed	426	5	1
Total SM	$(4.1\pm 0.3)\times 10^2$	$7.0\pm 1.2$	$1.3\pm 0.4$
$Z/\gamma ^*\rightarrow \ell \ell$	$209 \pm 18$	$4.6 \pm 1.0$	$0.9 \pm 0.3$
$Z\rightarrow \tau \tau$	$0.9 \pm 0.1$	$<$0.01	$<$0.01
$t\bar{t}$	$172 \pm 18$	$1.7 \pm 0.6$	$0.18 \pm 0.11$
Single top	$14 \pm 5$	$0.3 \pm 0.4$	$<$0.01
Diboson	$14 \pm 2$	$0.5 \pm 0.2$	$0.19 \pm 0.05$
Fake leptons (including $W$ $+$jets)	$<$0.01	$<$0.01	$<$0.01
$m_{\mathrm {LQ}}$ $=$ 300 GeV	$(12.0\pm 0.6)\times 10^3$	–	–
$m_{\mathrm {LQ}}$ $=$ 600 GeV	–	$152\pm 18$	–
$m_{\mathrm {LQ}}$ $=$ 1000 GeV	–	–	$3.4\pm 1.3$

The jet energy scale (JES) uncertainty depends on $p_{\mathrm {T}}$ and $\eta$ and contains additional factors, which are used to correct for pile-up effects. They are derived as a function of the number of primary vertices in the event to take into account additional *pp* collisions in a recorded event (in-time pile-up), or as a function of the expected number of interactions per bunch-crossing to constrain past and future collisions affecting the measurement of energies in the current bunch-crossing (out-of-time pile-up). An additional uncertainty on the jet energy resolution (JER) is taken into account. The relative impact on the background event yields from the JES (JER) uncertainty is between 8 % (1 %) in SR1 and 26 % (1 %) in SR8. The signal selection efficiency change due to the JES uncertainties ranges between 3 % in SR1 and 1 % in SR8, while the effect of the JER is negligible.

The electron energy scale and resolution are corrected to provide better agreement between MC predictions and data. The uncertainties on these corrections are propagated through the analysis as sources of systematic uncertainty. Uncertainties are taken into account for the electron trigger ($\sim$0.1 %), identification ($\sim$1 %) and reconstruction ($\sim$1 %) efficiencies, and for uncertainties associated with the isolation requirements ($\sim$0.1 %).

Scaling and smearing corrections are applied to the $p_{\mathrm {T}}$ of the muons in order to minimise the differences in resolution between data and MC simulated events. The uncertainty on these corrections is below 1 %. Differences in the identification efficiency and in the efficiency of the trigger selection are taken into account and are less than 1 %.

QCD renormalisation and factorisation scales are varied by a factor of two to estimate the impact of higher orders on the signal production cross-section. The variation is found to be approximately 14 % for all mass points. The uncertainty on the signal cross-section related to the choice of PDF set is evaluated as the envelope of the prediction of 40 different CTEQ6.6 NLO error sets [24]. The uncertainty ranges from 18 % at $m_{\mathrm {LQ}} = 300$ GeV to 56 % at $m_{\mathrm {LQ}} = 1300$ GeV. These uncertainties are the same for all LQ generations. The effect on the choice of PDF set on the signal acceptance times reconstruction efficiency is estimated using the Hessian method [59]. The final PDF uncertainties on the signal samples are approximately 1 % for most mass points, rising to 4 % for some higher LQ masses. The impact of the choice of PDF set on the acceptance times reconstruction efficiency for each background process is estimated using the Hessian method (using the same method as for signals). The uncertainties range from 4 % in the low-mass signal regions to 17 % in the high-mass signal regions.

### Results

The observed and expected yields in three representative signal regions for the *eejj* and the $\mu \mu$*jj* channels after the combined maximum likelihood fits are shown in Tables 2 and 3, respectively. The fit maximizes the likelihood constructed using the two CRs and the SR under study. When contructing the likelihood, the signal stregth and the background scale factors are treated as free parameters, the systematic uncertainties are treated as nuisance parameters.

No significant excess above the SM expectation is observed in any of the signal regions and a modified frequentist ${\mathrm {CL}}_s$ method [60] is used to set limits on the strength of the LQ signal, by constructing a profile likelihood ratio. Pseudo-experiments are used to determine the limits.

The cross-section limits on scalar LQ pair-production are presented as a function of $$\beta $$ for both channels in Fig. . Also shown are the results of the ATLAS searches for first- and second-generation LQs using 1.03 fb$$^{-1}$$ data at $$\sqrt{s}$$$$=$$ 7 TeV which also included searches in the *e*$$\nu $$*jj* and $$\mu \nu $$*jj* decay channels and therefore provide better sensitivity at low values of $$\beta $$. First (second)-generation scalar LQs are excluded for $$\beta =1$$ at 95 % CL for $$m_{\mathrm {LQ1}}< $$ 1050 GeV ($$m_{\mathrm {LQ2}}<$$ 1000 GeV). The expected exclusion ranges are the same as the observed ones. First (second)-generation scalar LQs are excluded for $$m_{\mathrm {LQ1}}<650$$ GeV ($$m_{\mathrm {LQ2}}<650$$ GeV) at $$\beta =0.2$$ and $$m_{\mathrm {LQ1}}<900$$ GeV ($$m_{\mathrm {LQ2}}<850$$ GeV) at $$\beta =0.5$$.

**Fig. 4:**
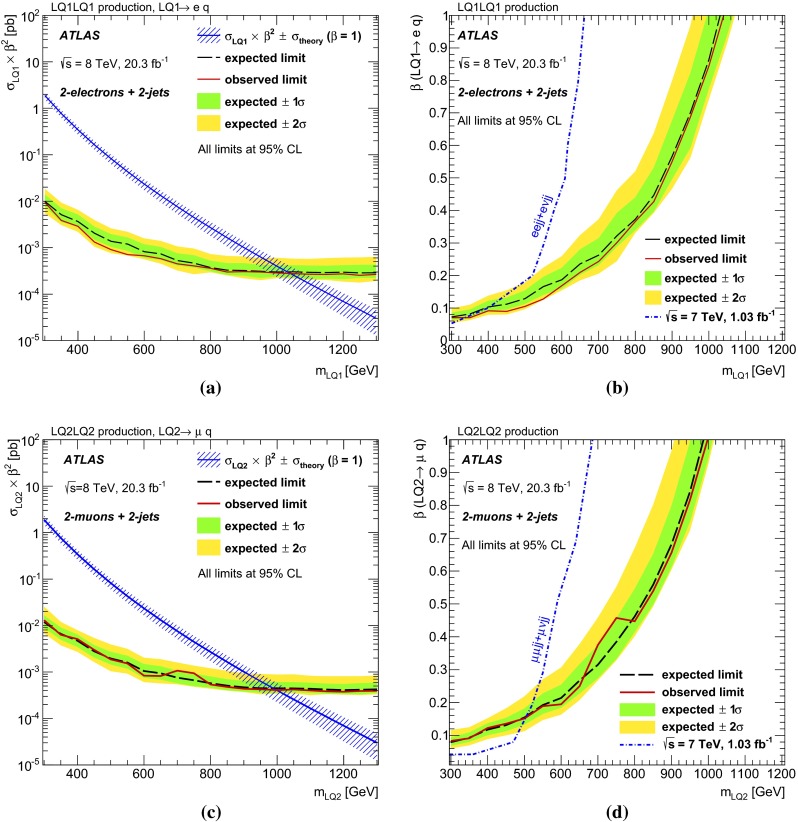
The cross-section limits on scalar LQ pair-production times the square of the branching ratio as a function of mass (*left*) and the excluded branching ratio as a function of the LQ mass (*right*) to *eq* for the *eejj* channel (*top*) and to $\mu q$ for the $\mu \mu$ *jj* channel (*bottom*). The $\pm 1 (2) \sigma$ uncertainty bands on the expected limit represent all sources of systematic and statistical uncertainty. The expected NLO production cross-section ($\beta = 1.0$) for scalar LQ pair-production and its corresponding theoretical uncertainty due to the choice of PDF set and renormalisation/factorisation scale are also included. The exclusion limits on $\mathrm {LQ}1$ [12] and $\mathrm {LQ}2$ [13] set by ATLAS in the *eejj* $+$ *e* $\nu$ *jj* and $\mu \mu$ *jj* $+$ $\mu \nu$ *jj* search channels using 1.03 fb$^{-1}$ of data collected at $\sqrt{s}=7$ TeV are also shown

**Table 4:**
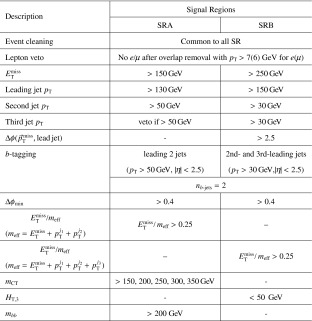
Summary of the event selection in each signal region for the $b\nu _{\tau }\bar{b}\bar{\nu _{\tau }}$ channel [18]

## Search for third-generation LQs in the $\varvec{b\nu _{\tau }\bar{b}\bar{\nu _{\tau }}}$ channel

The ATLAS search for pair-production of third-generation supersymmetric partners of bottom quarks (sbottom, $$\tilde{b}$$) [18] is reinterpreted in terms of the LQ model, in the case where each LQ decays to a *b*-quark and a $$\nu _{\tau }$$ neutrino. In the original analysis, the $$\tilde{b}$$ is assumed to decay via $$\tilde{b}$$$$\rightarrow b \tilde{\chi }^{0}$$, and $$\tilde{t}$$ via $$\tilde{t}$$$$\rightarrow b \tilde{\chi }^{\pm }$$ in the case where $$m_{\tilde{\chi }^{\pm }} - m_{\tilde{\chi }^{0}}$$ is small and the $$\tilde{\chi }^{\pm }$$ decay products are undetectable. The search is performed for final states with large missing transverse momentum ($$\mathbf {p}_{\mathrm {T}}^{\mathrm {miss}}$$, with magnitude $$E_{\mathrm {T}}^{\mathrm {miss}}$$) and two jets identified as originating from *b*-quarks. The full analysis strategy is covered in Ref. [18]. A complete description of the analysis, including treatment of systematic uncertainties on background processes can be found there, but the event selection and background estimation methods used are summarised here for clarity.

**Table 5:** For each signal region in the $b\nu _{\tau }\bar{b}\bar{\nu _{\tau }}$ channel, the observed event yield is compared with the background prediction obtained from the fit. Signal yields for different values of $m_{\mathrm {LQ}}$ (assuming $\beta = 0.0$) are given for comparison. The category ‘Others’ includes the diboson and $t\bar{t}$+*W* / *Z* processes. Statistical, detector-related and theoretical systematic uncertainties are included, taking into account correlations [18]

	SRA, $m_\mathrm {CT}$ $>$					SRB
	150 GeV	200 GeV	250 GeV	300 GeV	350 GeV	
Observed	102	48	14	7	3	65
Total SM	$94\pm 13$	$39\pm 6$	$16\pm 3$	$5.9\pm 1.1$	$2.5\pm 0.6$	$64 \pm 10$
Top quark	$11.1 \pm 1.8$	$2.4 \pm 1.4$	$0.4 \pm 0.3$	$<$0.01	$<$0.01	$41\pm 7$
*Z* production	$66 \pm 11$	$28 \pm 5$	$11 \pm 2$	$4.7 \pm 0.9$	$1.9 \pm 0.4$	$13\pm 4$
*W* production	$13 \pm 6$	$5 \pm 3$	$2.1 \pm 1.1$	$1.0 \pm 0.5$	$0.5 \pm 0.3$	$8\pm 5$
Others	$4.3 \pm 1.5$	$3.4 \pm 1.3$	$1.8 \pm 0.6$	$0.12 \pm 0.11$	$0.10_{-0.10}^{+0.12}$	$2.0\pm 1.0$
Multi-jet	$0.2 \pm 0.2$	$0.06 \pm 0.06$	$0.02 \pm 0.02$	$<$0.01	$<$0.01	$0.16\pm 0.16$
$m_{\mathrm {LQ}} = 300$ GeV	$(8.5\pm 0.2)\times 10^2$	$435\pm 17$	$96\pm 8$	$7\pm 2$	$0.6\pm 0.6$	$68\pm 7$
$m_{\mathrm {LQ}} = 600$ GeV	$21.9\pm 0.4$	$19.0\pm 0.4$	$15.6\pm 0.4$	$12.0\pm 0.3$	$8.7\pm 0.3$	$1.8\pm 0.1$

### Object and event selection

Events are required to have exactly two *b*-tagged [61] jets with $$p_{\mathrm {T}}$$ $$>$$ 20 GeV and $$|\eta |$$ $$<$$ 2.5, and $$E_{\mathrm {T}}^{\mathrm {miss}}$$$$>$$ 150 GeV. Additional jets in the event are accepted if they have $$p_{\mathrm {T}}$$$$>$$ 20 GeV and $$|\eta |$$ $$<$$ 4.9. Events with one or more electrons (muons) with $$p_{\mathrm {T}}$$ $$>$$ 7 (6) GeV are vetoed. Candidate signal events are selected from data using a $$E_{\mathrm {T}}^{\mathrm {miss}}$$ trigger which is 99 % efficient for events passing the offline selection. Several variables are defined and used to optimise the event selection:

- $\mathrm {\Delta }\phi _{\mathrm {min}}$: The minimum azimuthal distance ($\Delta \phi$) between any of the leading three jets and the $\vec {p}_\mathrm {T}^{\mathrm {\,miss}}$.
- $m_{\mathrm {eff}}$: The scalar sum of the $p_{\mathrm {T}}$ of the leading two or three jets (depending on the signal region) and the $E_{\mathrm {T}}^{\mathrm {miss}}$.
- $H_{\mathrm{T},3}$: The scalar sum of the $p_{\mathrm {T}}$ of all but the leading three jets.
- $$m_{bb} $$: The invariant mass of the two $$b$$-tagged jets in the event.
- $m_\mathrm {CT}$: The contransverse mass [62], used to measure the masses of pair-produced heavy particles that decay semi-invisibly (i.e. decays where one of the decay products can be detected, but the other cannot).

In the original analysis, different signal regions were optimised according to the masses of the third-generation squark and the lightest supersymmetric particle (LSP). In the case of the LQ model reinterpretation the signal regions corresponding to the case where the mass of the LSP is approximately zero have best sensitivity, but all the signal regions are retained for coherence with the original analysis. The different signal region definitions are given in Table 4. Signal region A (SRA) has five different $m_\mathrm {CT}$ thresholds. Signal region B (SRB) is optimised towards the region where the squark and LSP masses are approximately equal. The signal region with the best expected limit is used for each point in the exclusion plots.

### Background estimation

The dominant background process is the production of *Z* bosons in association with heavy-flavour jets where the *Z* boson subsequently decays to two neutrinos [$$Z(\rightarrow \nu \nu )+b\bar{b}$$]. Its contribution is estimated from data in an opposite-sign dilepton control region. Top quark pair-production ($$t\bar{t}$$) and *W* bosons produced in association with heavy flavour quarks also contribute significantly and are normalised in dedicated control regions before being extrapolated to the signal regions using MC simulation. Different control regions are defined for each signal region, requiring one or two leptons plus additional requirements similar to the corresponding signal region. The contributions from $$Z$$$$+$$jets, *W*+jets, and top quark production are estimated simultaneously with a profile likelihood fit to the three control regions. Contributions from diboson and $$t\bar{t}$$+*W* / *Z* processes are estimated from MC simulation in all regions. The contribution from multi-jet events is estimated from data by taking well-measured multi-jet events from data and smearing the jets with jet response functions taken from MC simulation and validated in data. This procedure is described in detail in Ref. [63]. The contribution from multi-jet events in signal regions is found to be negligible.

### Results

The number of data events observed in each signal region is reported in Table 5, together with the SM background expectation after the background-only fit, and the expected number of signal events for different LQ masses. The signal acceptance efficiency is around 2 % for all but the lowest LQ masses targeted (dropping to 0.27 % efficiency for $m_{\mathrm {LQ}}$ $=$ 200 GeV). All sources of systematic and statistical uncertainty are taken into account. The dominant systematic uncertainties on the background prediction are the jet energy scale (JES 1–5 %) and resolution (JER 1–8 %), and the *b*-tagging uncertainty (2–10 %). Detector-related systematic uncertainties on the signal prediction are dominated by uncertainties on the *b*-tagging efficiency ($\sim$30 %). The second-largest source of uncertainty is due to the JES and is around 3 %.

The uncertainties on the signal production cross-section are estimated using the methods described in Sect. 4.6. These uncertainties are the same for all LQ genertions but the uncertainty due to the choice of PDF set varies with $m_{\mathrm {LQ}}$. Since the third-generation analyses consider a different mass range to the first- and second-generation analyses, in this case the uncertainty due to the choice of PDF set ranges from 7.1 % at $m_{\mathrm {LQ}} = 200$ GeV to 30 % at $m_{\mathrm {LQ}} = 800$ GeV. Effects on the acceptance due to the choice of PDF set are negligible.

No significant excess above the SM expectation is observed in any of the signal regions. Figure  shows the observed and expected exclusion limits for the scalar $\mathrm {LQ}$3 pair-production scenario obtained by taking, for each signal mass configuration, the signal region with the best expected limit. These limits are obtained using the methods described in Sect. 4.7. These methods compare the observed numbers of events in the signal regions with the fitted background expectation and accounting for signal contamination in the corresponding CRs for a given model. Pair-produced third-generation scalar LQs decaying to $b\nu _{\tau }\bar{b}\bar{\nu _{\tau }}$ are excluded at 95 % CL for $m_{\mathrm {LQ3}}$ $<$ 625 GeV. The expected excluded range is $m_{\mathrm {LQ3}}$ $<$ 640 GeV.

**Fig. 5:**
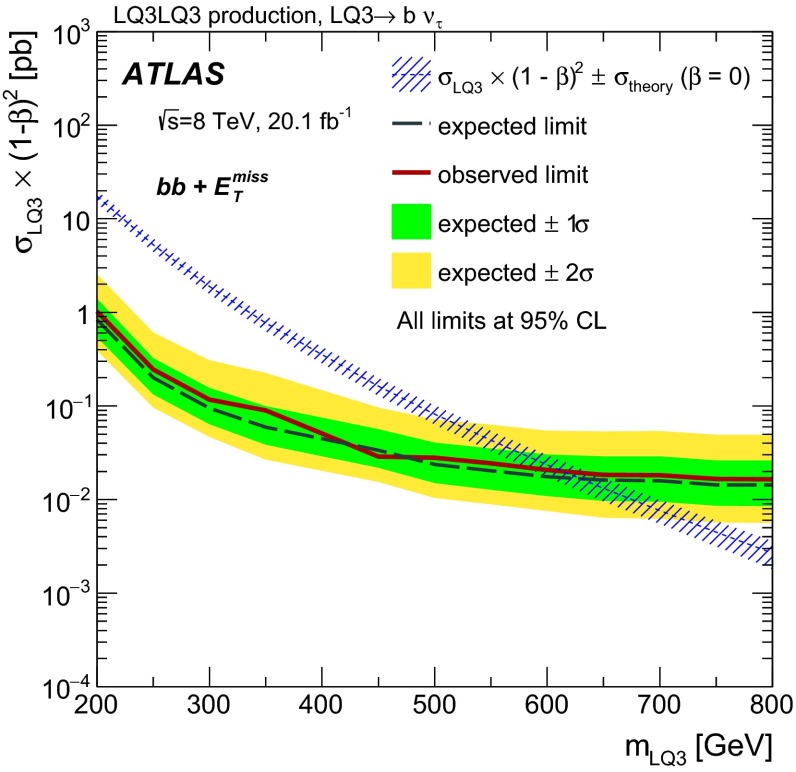
The expected (*dashed*) and observed (*solid*) 95 % CL upper limits on third-generation scalar LQ pair-production cross-section times the square of the branching ratio to $b\nu _{\tau }$ as a function of LQ mass, for the $b\nu _{\tau }\bar{b}\bar{\nu _{\tau }}$ channel. The $\pm 1 (2) \sigma$ uncertainty bands on the expected limit represent all sources of systematic and statistical uncertainty. The expected NLO production cross-section ($\beta = 0.0$) for scalar LQ pair-production and its corresponding theoretical uncertainty due to the choice of PDF set and renormalisation/factorisation scale are also included

## Search for third-generation LQs in the $\varvec{t\nu _{\tau }\bar{t}\bar{\nu _{\tau }}}$ channel

The ATLAS search for pair-production of the supersymmetric partner of the top quark (stop quark, $\tilde{t}$) [19] is reinterpreted in terms of the LQ model, in the case where each LQ decays to a top quark and a $\nu _{\tau }$ neutrino. The original analysis has dedicated signal regions targeting $\tilde{t}$ decays into $t \tilde{\chi }^{0}$ and the subsequent semileptonic decay of the $t\bar{t}$ pair. Events compatible with $t\bar{t}$ plus extra $E_{\mathrm {T}}^{\mathrm {miss}}$ are selected with final states containing one isolated lepton, jets, and $E_{\mathrm {T}}^{\mathrm {miss}}$. A complete description of the analysis strategy, including the treatment of systematic uncertainties on background processes can be found in Ref. [19]. The event selection and the background estimation methods are summarised here for clarity.

**Table 6:**
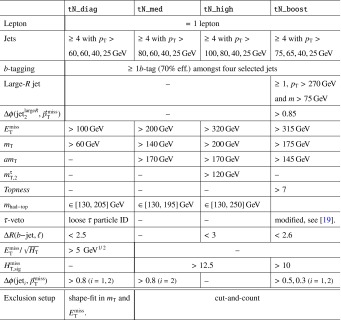
Selection criteria for the four SRs (tN_diag, tN_med, tN_high, and tN_boost) employed to search for $\mathrm {LQ}3\,\overline{\mathrm {LQ}3}\rightarrow t\nu _{\tau }\bar{t}\bar{\nu _{\tau }}$ events [19]. The details of the limit-setting procedure for the exclusion setup can be found in Sect. 6.3

### Object and event selection

Events are required to contain exactly one electron with $p_{\mathrm {T}}$ $>$ 25 GeV and $|\eta |$ $<$ 2.47, or muon with $p_{\mathrm {T}}$ $>$ 25 GeV and $|\eta |$ $<$ 2.4. Events containing more than one electron or muon with looser identification and $p_{\mathrm {T}}$ requirements (10 GeV for both) are vetoed. In some signal regions, events are vetoed if they are consistent with containing a hadronically decaying $\tau$ lepton. Events are required to have a minimum of four jets with $p_{\mathrm {T}}$ $>$ 20 GeV and $|\eta |$ $<$ 2.5, with at least one of these passing *b*-tagging requirements [61]. In addition, selected events must have $E_{\mathrm {T}}^{\mathrm {miss}}$ $>$ 100 GeV. Several variables are used to further select signal events and reject background processes:

- $m_{\mathrm {T}}$: The transverse mass of the electron or muon and the $E_{\mathrm {T}}^{\mathrm {miss}}$.
- $am_{\mathrm{T}}$ and $m_{\mathrm{T},2}^{\tau }$: These are two variants on the stransverse mass ($m_{\mathrm{T},2}$) [64–66] which is a generalisation of the transverse mass when applied to signatures with two invisible particles in the final state. The asymmetric stransverse mass $am_{\mathrm{T}}$, aims to reject dileptonic $t\bar{t}$ events where one of the leptons is not reconstructed or is outside the acceptance (and therefore adds to the $E_{\mathrm {T}}^{\mathrm {miss}}$ of the event). The second implementation of this variable, the $\tau$ stransverse mass $m_{\mathrm{T},2} ^\tau$, targets $t\bar{t}$ events where one top decays leptonically and the other top decays into a $\tau$ that subsequently decays hadronically.
- *topness*: This variable is designed to reject dileptonic $t\bar{t}$ events where one lepton is assumed to be lost, as detailed in Ref. [67]. The *topness* variable is based on the minimisation of a $\chi ^{2}$-type function.
- $m_\mathrm{{had-top}}$: This quantity is used to reject dileptonic $t\bar{t}$ events but retain signal events that contain a hadronically decaying on-shell top quark, as in the $\mathrm {LQ}\rightarrow t + \nu _{\tau }$ and $\tilde{t}^{}_{1} \rightarrow t \tilde{\chi }^0_1$ scenarios.
- $\Delta \phi (\text {jet}_{1,2}, \vec {p}_\mathrm {T}^{\mathrm {\,miss}})$: The azimuthal opening angle between the leading or sub-leading jet and $\vec {p}_\mathrm {T}^{\mathrm {\,miss}}$ used to suppress multi-jet events where $\vec {p}_\mathrm {T}^{\mathrm {\,miss}}$ is aligned with one of the leading two jets.
- $\mathrm {MetSig}$: An approximation of the $E_{\mathrm {T}}^{\mathrm {miss}}$ significance, where $H_\mathrm {T}$ is defined as the scalar $p_{\mathrm {T}}$ sum of the leading four jets.
- $H_\mathrm {T,sig}^{\mathrm {miss}}$: An object-based missing transverse momentum, divided by the per-event resolution of the jets, and shifted to the scale of the background [68].

**Table 7:** The number of observed events in the three cut-and-count signal regions, together with the expected number of background events and signal events for different $\mathrm {LQ}$ masses (assuming $\beta = 0.0$) in the $t\nu _{\tau }\bar{t}\bar{\nu _{\tau }}$ channel [19]

	tN_med	tN_high	tN_boost
Observed	12	5	5
Total SM	13 $\pm$ 2	5.0 $\pm$ 0.9	3.3 $\pm$ 0.7
$t\bar{t}$	6.5 $\pm$ 1.7	2.0 $\pm$ 0.6	1.1 $\pm$ 0.4
$W$ $+$jets	2.1 $\pm$ 0.5	0.9 $\pm$ 0.3	0.28 $\pm$ 0.14
Single top	1.1 $\pm$ 0.5	0.54 $\pm$ 0.19	0.39 $\pm$ 0.15
Diboson	1.4 $\pm$ 0.6	0.9 $\pm$ 0.3	0.7 $\pm$ 0.3
$Z$ $+$jets	0.009 $\pm$ 0.005	0.003 $\pm$ 0.002	0.004 $\pm$ 0.002
$t\bar{t}$ *V*	2.0 $\pm$ 0.6	0.8 $\pm$ 0.3	0.9 $\pm$ 0.3
$m_{\mathrm {LQ}} = 300$ GeV	20 $\pm$ 3	3.4 $\pm$ 1.1	3.8 $\pm$ 1.2
$m_{\mathrm {LQ}}= 600$ GeV	10.7 $\pm$ 0.3	7.9 $\pm$ 0.3	8.9 $\pm$ 0.3

**Table 8:** The number of observed events in the shape-fit signal region, together with the expected number of background events and signal events for different $\mathrm {LQ}$ masses (assuming $\beta = 0.0$) in the $t\nu _{\tau }\bar{t}\bar{\nu _{\tau }}$ channel [19]

	tN_diag			
	$125 < E_{\mathrm {T}}^{\mathrm {miss}}< 150$ GeV	$125 < E_{\mathrm {T}}^{\mathrm {miss}}< 150$ GeV	$E_{\mathrm {T}}^{\mathrm {miss}}> 150$ GeV	$E_{\mathrm {T}}^{\mathrm {miss}}> 150$ GeV
	$120 < m_{\mathrm {T}} < 140$ GeV	$m_{\mathrm {T}} > 140$ GeV	$120 < m_{\mathrm {T}} < 140$ GeV	$m_{\mathrm {T}} > 140$ GeV
Observed	117	163	101	217
Total SM	$(1.4 \pm 0.2)\times 10^2$	$(1.5 \pm 0.2)\times 10^2$	98 $\pm$ 13	$(2.4 \pm 0.3)\times 10^2$
$t\bar{t}$	$(1.2 \pm 0.2)\times 10^2$	$(1.4 \pm 0.2)\times 10^2$	85 $\pm$ 12	($2.0 \pm 0.3)\times 10^2$
$W$ $+$jets	7 $\pm$ 3	6 $\pm$ 3	4.6 $\pm$ 1.5	10 $\pm$ 4
Single top	5 $\pm$ 2	6 $\pm$ 2	6$\pm$ 2	9 $\pm$ 4
Diboson	0.29 $\pm$ 0.18	0.8 $\pm$ 0.5	0.3 $\pm$ 0.3	0.30 $\pm$ 0.15
$Z$ $+$jets	0.17 $\pm$ 0.08	0.24 $\pm$ 0.12	0.30 $\pm$ 0.15	0.5 $\pm$ 0.3
$t\bar{t}$ *V*	1.5 $\pm$ 0.5	2.9 $\pm$ 0.9	2.5 $\pm$ 0.8	11 $\pm$ 3
$m_{\mathrm {LQ}} = 300$ GeV	28 $\pm$ 3	77 $\pm$ 6	64 $\pm$ 5	269 $\pm$ 10
$m_{\mathrm {LQ}} = 600$ GeV	0.15 $\pm$ 0.04	0.62 $\pm$ 0.08	0.83 $\pm$ 0.09	18.8 $\pm$ 0.4

The variables listed above are used to define three cut-and-count SRs and one shape-fit SR. Table 6 details the event selections for these signal regions. The SR labelled tN_boost targets LQ/stop masses of $\gtrsim$700 GeV and takes advantage of the ‘boosted’ topology of such a heavy parent particle. The selection assumes that either all decay products of the hadronically decaying top quark, or at least the decay products of the hadronically decaying *W* boson, collimate into a jet reconstructed with a radius parameter $R = 1.0$ [69, 70].

### Background estimation

The dominant sources of background are the production of $$t\bar{t}$$ events and $$W$$$$+$$jets where the *W* boson decays leptonically. Other background processes considered are single top, dibosons, $$Z$$$$+$$jets, $$t\bar{t}$$ produced in association with a vector boson ($$t\bar{t}$$*V*), and multi-jets.

The predicted numbers of $$t\bar{t}$$ and $$W$$$$+$$jets background events in the SRs are estimated from data using a fit to the number of observed events in dedicated control regions. Each SR has an associated CR for each of the $$t\bar{t}$$ and $$W$$$$+$$jets backgrounds. The CRs are designed to select events as similar as possible to those selected by the corresponding SR while keeping the contamination from other backgrounds and potential signal low. This is achieved by e.g. requiring that 60 $$<$$ $$m_{\mathrm {T}} $$$$<$$ 90 GeV and in the case of the $$W$$$$+$$jets CR, inverting the *b*-jet requirement so that it becomes a *b*-jet veto. The simulation is used to extrapolate the background predictions into the signal region. The background fit predictions are validated using dedicated event samples, referred to as validation regions (VRs), and one or more VR is defined for each of these. Most VRs are defined by changing the $$m_{\mathrm {T}} $$ windows to 90 $$<$$ $$m_{\mathrm {T}} $$$$<$$ 120 GeV. The VRs are designed to be kinematically close to the associated SRs to test the background estimates in regions of phase space as similar as possible to the SRs.

The multi-jet background is estimated from data using a matrix method described in Refs. [71, 72]. The contribution is found to be negligible. All other (small) backgrounds are determined entirely from simulation and normalised to the most accurate theoretical cross-sections available.

### Results

The number of events observed in each signal region is reported in Tables 7 and 8, together with the SM background expectation and the expected number of signal events for different LQ masses. The signal acceptance is between 1.5 and 3 % depending on the LQ mass. All sources of systematic uncertainty and statistical uncertainty are taken into account. The dominant sources of uncertainty on the background prediction come from uncertainties related to the JES, JER, $t\bar{t}$ background modelling, the *b*-tagging efficiency, and statistical uncertainties.

**Fig. 6:**
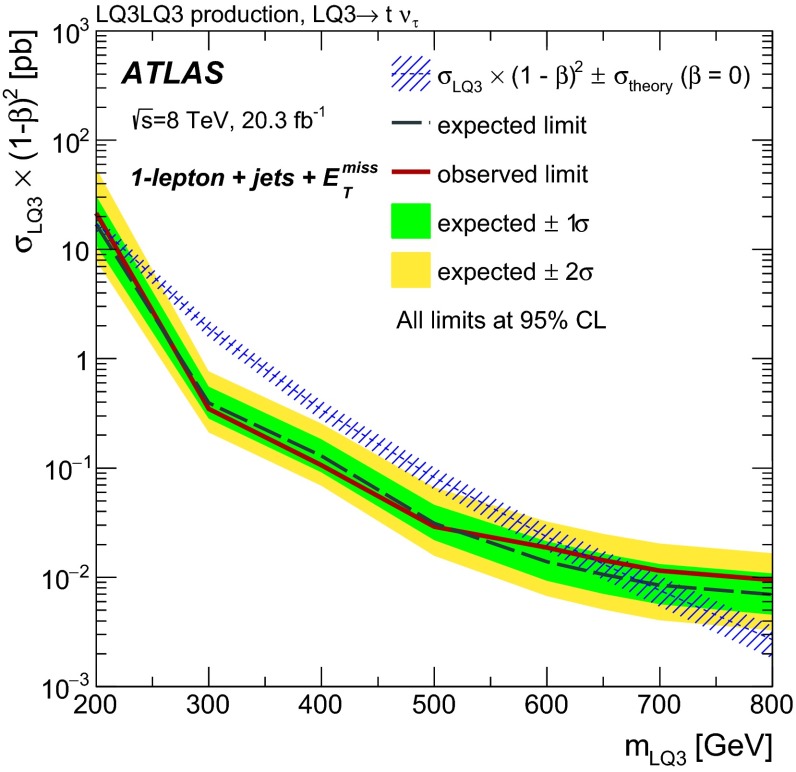
The expected (*dashed*) and observed (*solid*) 95 % CL upper limits on the third-generation scalar LQ pair-production cross-section times the square of the branching ratio to $t\nu _{\tau }$ as a function of LQ mass, for the $t\nu _{\tau }\bar{t}\bar{\nu _{\tau }}$ channel. The $\pm 1 (2) \sigma$ uncertainty bands on the expected limit represent all sources of systematic and statistical uncertainty. The expected NLO production cross-section ($\beta = 0.0$) for third-generation scalar LQ pair-production and its corresponding theoretical uncertainty due to the choice of PDF set and renormalisation/factorisation scale are also included

Detector-related systematic effects are evaluated for signal using the same methods used for the backgrounds (see Ref. [19] for details). The dominant detector-related systematic effects are the uncertainties on the JES (4 %) and the *b*-tagging efficiency (3 %).

The uncertainties on the signal production cross-section are estimated using the methods described in Sect. 4.6. The effect on the choice of PDF set on the signal acceptance is less than 1 % for most mass points, but increases to 1.7 % for $m_{\mathrm {LQ}} = 800$ GeV.

Similar methods as described in Sect. 4.7 are used to assess the compatibility of the SM background-only hypothesis with the observations in the signal regions. The observed number of events is found to agree well with the expected number of background events in all signal regions. No significant excess over the expected background from SM processes is observed and the data are used to derive one-sided limits at 95 % CL. The results are obtained from a profile likelihood-ratio test following the ${\mathrm {CL}}_s$ prescription [60]. The likelihood of the simultaneous fit is configured to include all CRs and one SR or shape-fit bin. The ‘exclusion setup’ event selection is applied (see Table 6), and all uncertainties except the theoretical signal uncertainty are included in the fit.

Exclusion limits are obtained by selecting a priori the signal region with the lowest expected ${\mathrm {CL}}_s$ value for each signal grid point. The expected and observed limits on the $\mathrm {LQ}3\,\overline{\mathrm {LQ}3}\rightarrow$ $t\nu _{\tau }\bar{t}\bar{\nu _{\tau }}$ process are shown in Fig. . Third-generation scalar LQs decaying to $t\nu _{\tau }\bar{t}\bar{\nu _{\tau }}$ are excluded at 95 % CL in the mass range 210 $<$ $m_{\mathrm {LQ3}}$ $<$ 640 GeV. The expected exclusion range is 200 $<$ $m_{\mathrm {LQ3}}$ $<$ 685 GeV. The limits for stop production in the case where the neutralino is massless are slightly stronger than the limits set on LQ3 production since the nominal stop limits consider a mostly right-handed stop. This leads to the top quarks being polarised in such a way that the acceptance increases. The limit worsens at low mass, due to the effect of greater contamination from top backgrounds.

## Summary and conclusions

Searches for pair-production of first-, second- and third-generation scalar leptoquarks have been performed with the ATLAS detector at the LHC using an integrated luminosity of 20 fb$^{-1}$ of data from *pp* collisions at $\sqrt{s} = 8$ TeV. No significant excess above the SM background expectation is observed in any channel. The results are summarised in Table 9.

**Table 9:** Expected and observed exclusion ranges at 95 % CL for each of the four LQ decay channels considered

Decay channel	Excluded range (95 % CL)	
	Expected	Observed
*eejj* ($\beta = 1.0$)	$m_{\mathrm {LQ1}}< 1050$ GeV	$m_{\mathrm {LQ1}}< 1050$ GeV
$\mu \mu$ *jj* ($\beta = 1.0$)	$m_{\mathrm {LQ2}}< 1000$ GeV	$m_{\mathrm {LQ2}}< 1000$ GeV
$b\nu _{\tau }\bar{b}\bar{\nu _{\tau }}$($\beta = 0.0$)	$m_{\mathrm {LQ3}} < 640$ GeV	$m_{\mathrm {LQ3}} < 625$ GeV
$t\nu _{\tau }\bar{t}\bar{\nu _{\tau }}$($\beta = 0.0$)	$200 < m_{\mathrm {LQ3}} < 685$ GeV	$210 < m_{\mathrm {LQ3}} < 640$ GeV

The results presented here significantly extend the sensitivity in mass compared to previous searches. Low-mass regions are also considered and limits on the cross-sections are provided for the different final states analysed. Since $\beta$ is not constrained by the theory, searches in the low mass regions are also important in order to extract limits for low-$\beta$ values for the $\mathrm {LQ}$1 and $\mathrm {LQ}$2 analyses.

## Footnotes

1 ATLAS uses a right-handed coordinate system with its origin at the nominal interaction point (IP) in the centre of the detector and the *z*-axis along the beam pipe. The *x*-axis points from the IP to the centre of the LHC ring, and the *y*-axis points upward. Cylindrical coordinates $(r,\phi )$ are used in the transverse plane, $\phi$ being the azimuthal angle around the beam pipe. The pseudorapidity is defined in terms of the polar angle $\theta$ as $\eta =-\ln \tan (\theta /2)$.
